# Spatial organization of *Clostridium difficile* S-layer biogenesis

**DOI:** 10.1038/s41598-020-71059-x

**Published:** 2020-08-24

**Authors:** Peter Oatley, Joseph A. Kirk, Shuwen Ma, Simon Jones, Robert P. Fagan

**Affiliations:** 1 Department of Molecular Biology and Biotechnology, Florey Institute, University of Sheffield, Sheffield, S10 2TN UK; 2 School of Medicine, University of Central Lancashire, Preston, PR1 7BH UK; 3 Department of Chemistry, University of Sheffield, Sheffield, S3 7HF UK

**Keywords:** Protein transport, Cellular microbiology

## Abstract

Surface layers (S-layers) are protective protein coats which form around all archaea and most bacterial cells. *Clostridium difficile* is a Gram-positive bacterium with an S-layer covering its peptidoglycan cell wall. The S-layer in *C. difficile* is constructed mainly of S-layer protein A (SlpA), which is a key virulence factor and an absolute requirement for disease. S-layer biogenesis is a complex multi-step process, disruption of which has severe consequences for the bacterium. We examined the subcellular localization of SlpA secretion and S-layer growth; observing formation of S-layer at specific sites that coincide with cell wall synthesis, while the secretion of SlpA from the cell is relatively delocalized. We conclude that this delocalized secretion of SlpA leads to a pool of precursor in the cell wall which is available to repair openings in the S-layer formed during cell growth or following damage.

## Introduction

**Figure 1:**
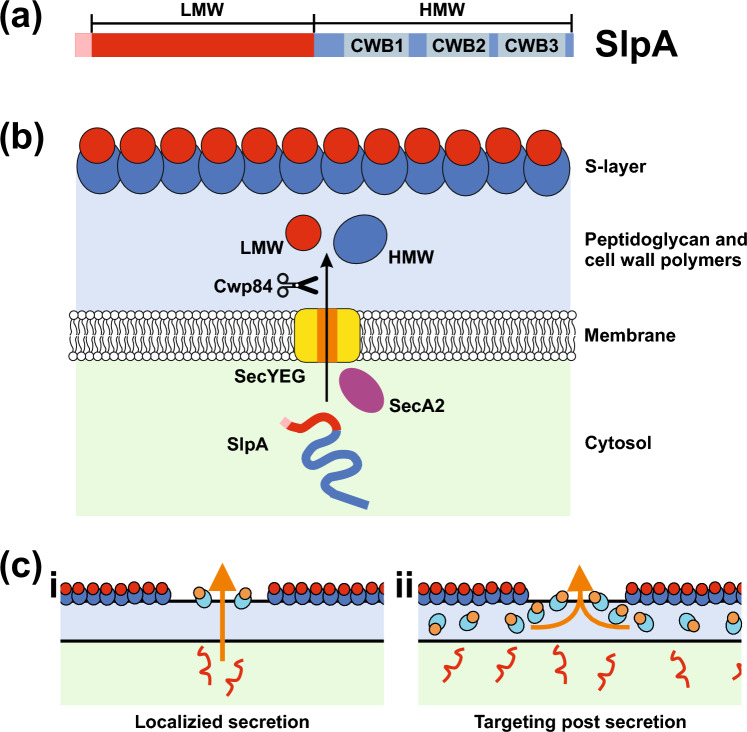
The *C. difficile* S-layer and the SlpA secretory pathway. (**a**) Domain structure of SlpA precursor protein with signal sequence (Pink), low molecular weight region (LMW, Red) and high molecular weight region (HMW, Blue) that contains three cell wall binding domains (CWB, 1–3 in Grey). (**b**) Schematic diagram of SlpA secretion and processing in *C. difficile*. SlpA (Pink/Red/blue line) is translated in the cytosol (light green) and targeted for secretion across the membrane using SecA2 (Purple oval) most likely via the SecYEG Channel (Yellow/Orange). Cwp84 (Scissors) cleaves SlpA into low molecular weight (LMW, Red spheres) and high molecular weight (HMW, Blue spheres) S-layer protein (SLP) subunits. The HMW and LMW SLPs assemble to form hetero-dimers that incorporate into the S-layer. The surface of the S-layer consists largely of exposed LMW-SLP anchored to the cell wall (light blue) via cell wall biding domains of the HMW-SLP component (see (**a**)). (**c**) Models of SlpA integration into the S-layer. (i) unfolded SlpA (red line) is secreted from specific points on the cell membrane—directed by gaps in the S-layer or cell wall (colored as in (**b**)), newly processed SlpA (LMW-SLP orange circles, HMW-SLP light blue ovals) is transported directly through the cell wall for integration into the S-layer. Alternatively; (ii) SlpA is translocated across the cell membrane at multiple sites. A pool of SlpA lays within the cell wall ready to fill gaps in the S-layer.

*Clostridium difficile* infection (CDI) is the major cause of antibiotic associated diarrhoea^1^ and can lead to severe inflammatory complications^2^. This Gram-positive bacterium has a cell wall encapsulating, proteinaceous surface-layer (S-layer), a paracrystalline array that acts as a protective semipermeable shell and is essential for virulence^3^. In *C. difficile* the S-layer largely consists of SlpA, the most abundant surface protein, with additional functionality added through the incorporation of up to 28 minor S-layer-associated cell wall proteins^4^. SlpA is produced as a pre-protein (Fig. 1a) that is secreted and processed by the cell surface cysteine protease Cwp84 into low molecular weight (LMW) and high molecular weight (HMW) SLP subunits^5^ (Fig. 1b). These two subunits form a heterodimeric complex that is then incorporated into the crystalline lattice of the S-layer, which is anchored to cell wall polysaccharide PS-II via three cell wall binding (CWB2) motifs within the HMW region^6,7^ (Fig. 1a).

The production and secretion of S-layer components are energetically expensive for the cell, suggesting that the process will display evolved efficiency. However, it is not yet clear how S-layer formation is spatially regulated and whether SlpA is targeted to areas of cellular growth before or after secretion (Fig. 1c). *C. difficile* express two homologs of the *E. coli* cytosolic protein export ATPase, SecA: SecA1 and SecA2^8^. These two SecAs are thought to promote post-translational secretion through the general secretory (Sec) pathway. SecA2 is required for efficient SlpA secretion^8^ and is encoded adjacent to *slpA* on the chromosome^9^. It has been shown that some SecA2 systems secrete specific substrates (reviewed by^10^) which may ease the burden on the general Sec system and allow spatial or temporal regulation of secretion.

As an obligate anaerobe, *C. difficile* has been notoriously difficult to visualize using standard microscopy techniques with commonly used oxygen-dependent fluorescent proteins and this is further complicated by intrinsic autofluorescence in the green spectrum^11^. To circumvent these problems, we have used a variety of labeling techniques to avoid the requirement for oxygen maturation and any overlap with autofluorescence. Using fluorescence microscopy, we identified areas of S-layer biogenesis and SlpA secretion to determine if this S-layer component is specifically targeted to growing parts of the cell. Firstly, we probed the localization of newly synthesized S-layer which was detected at discrete regions which coincided with areas of new peptidoglycan biosynthesis. We continued by studying the internal localization of SecA2 and SlpA, discovering that SlpA is secreted all over the cytoplasmic membrane. Having observed delocalized secretion of SlpA, yet localized new surface S-layer, we conclude that there is a pool of SlpA that resides within the cell wall which is available to construct regions of the developing S-layer.

## Results

### Newly synthesized S-layer co-localizes with areas of new peptidoglycan

**Figure 2:**
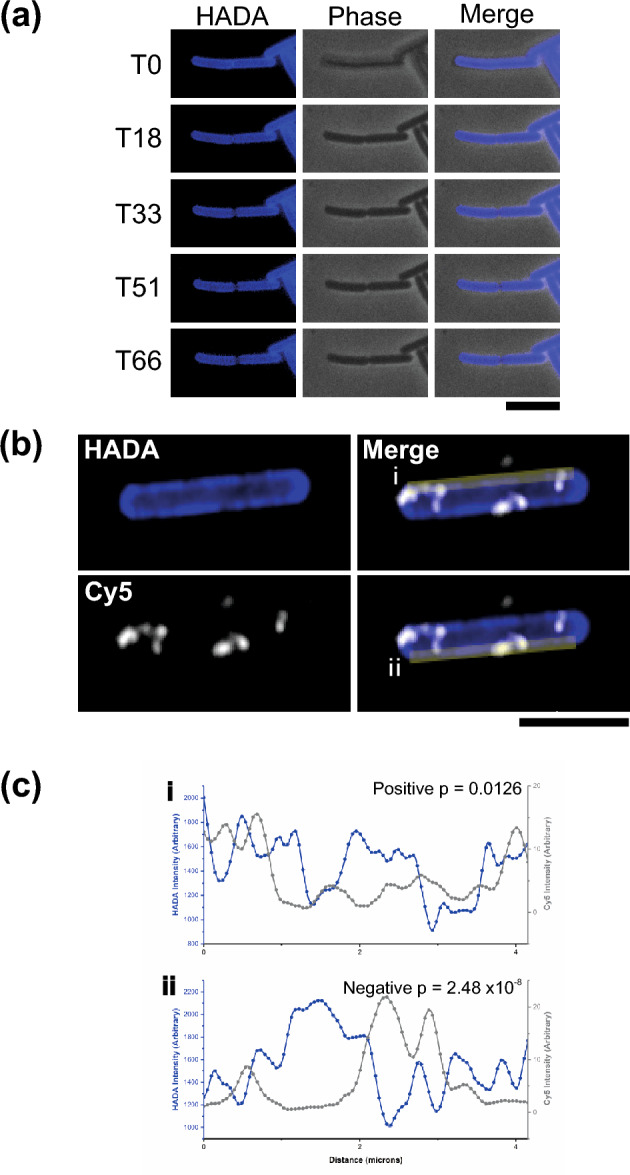
New surface S-layer colocalizes with areas of new peptidoglycan synthesis. (**a**) Examples of timepoints from real-time widefield fluorescent HADA signal (left panels) and phase contrast (center panels) of *C. difficile* 630 cells chased for HADA stain. Frame time represented in minutes, scale bar indicates 6 µm. (**b**) Airyscan confocal image of a *C. difficile* 630 cell grown with HADA to label peptidoglycan (Blue) and chased to reveal darker areas of newly synthesized peptidoglycan in the cell wall. This chase was followed by a short expression of SlpA_R20291_ which was specifically immunolabeled with Cy5 (White). After merging HADA and Cy5 channels (righthand panels, duplicated for clarity), cell sides (yellow bars) were selected for signal intensity analysis. For this example cell the intensity plots for cell sides (i) and (ii) are shown in panel C. Scale bar indicates 6 µm. (**c**) Intensity plot depicting signal from HADA (Blue) and Cy5 (Grey) along the yellow bars illustrated in (**b**). Trace (i) was calculated to have a significant positive correlation and (ii) a significant negative correlation.

During exponential growth, *C. difficile* cells are constantly growing and dividing, requiring the production of new peptidoglycan at the cell wall. The S-layer protects the cell envelope from innate immune effectors such as lysozyme and LL-37^3^. This function requires that an S-layer barrier is maintained while new peptidoglycan is synthesized during growth. Peptidoglycan can be labelled by growing *C. difficile* cells in the presence of the fluorescent d-amino acid, HCC-amino-d-alanine (HADA)^12^. Subsequent chasing with unlabeled media and imaging of live cells (Fig. 2a and Supplementary Movie 1) or fixed cells over a time course (Supplementary Fig. 1) reveals sites of newly synthesized peptidoglycan that appear less intense for HADA. This pattern of HADA staining is seen at the dividing septum and along the long axis of the cell (Fig. 2). Combining this with the inducible expression of the immunologically distinct SlpA_R20291_ in *C. difficile* strain 630 (Supplementary Fig. 2) and the impermeability of the S-layer to large molecules^3^, allowed areas of newly assembled S-layer at the surface to be visualized by immunofluorescence (Fig. 2b and Supplementary Fig. 2). To determine if the appearance of new SlpA exposed on the cell surface correlated with new peptidoglycan synthesis, and hence cell growth, we performed correlation analyses between the HADA and Cy5 signals along the long axis cell boundary (Fig. 2b,c, Supplementary Table S1). Of 76 cell sides analyzed, 50 displayed a negative correlation, of which 26 were statistically significant. In contrast only 26 sides displayed an apparent positive correlation, with only 6 being significant. An example cell showing a clear and significant negative correlation on one side and positive correlation on the other is shown in Fig. 2, additional examples are shown in Supplementary Fig. 3. This indicates that new S-layer formation is most likely at sites of peptidoglycan synthesis and cell growth. The areas of newly synthesized peptidoglycan that are void of SlpA_R20291_ signal are likely to be filled with endogenous SlpA_630_ that is expressed at much higher levels, as observed in extracellular cell wall protein extracts (Supplementary Fig. 2). However, we cannot rule out that these areas lacking SlpA signal were inaccessible to antibody due to cell aggregation during staining.

**Figure 3:**
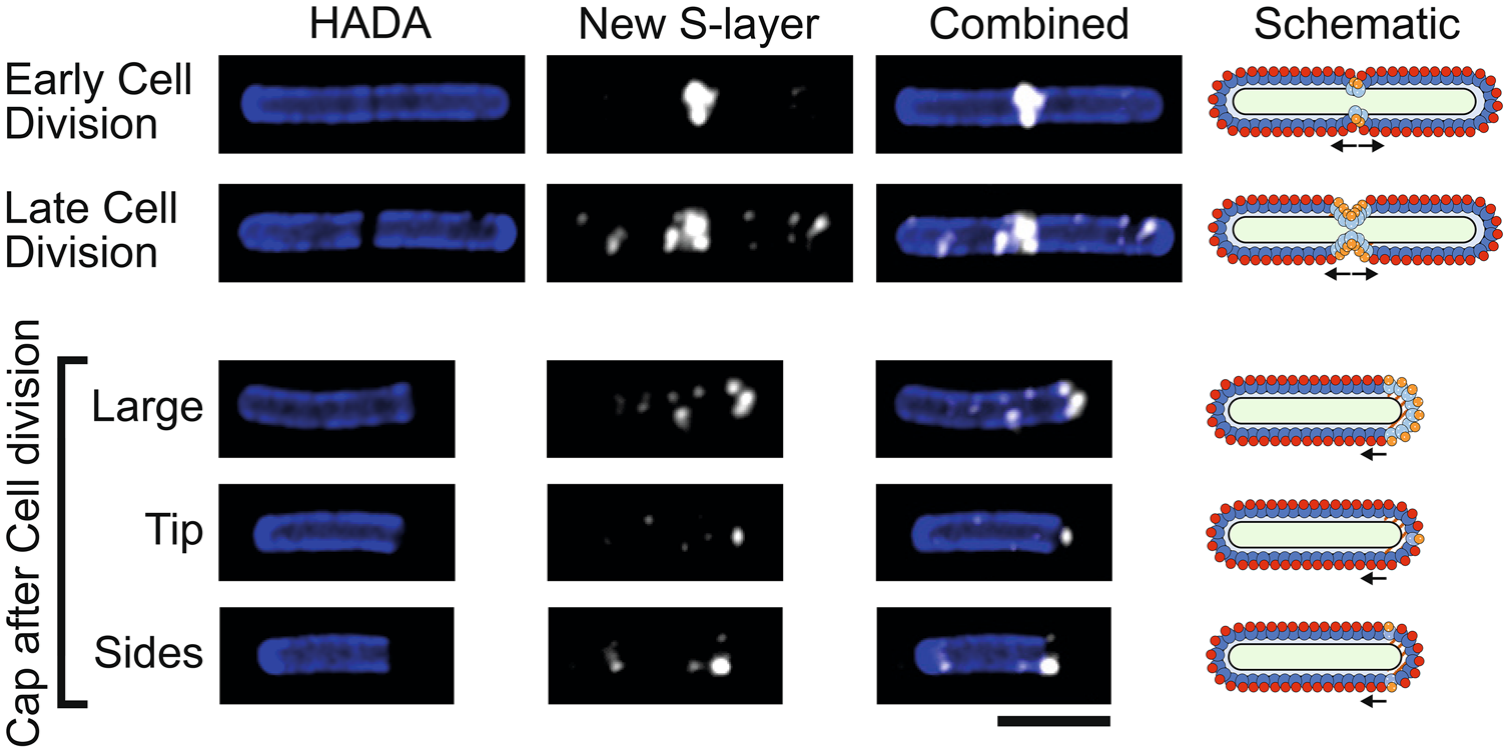
S-layer formation during cell division. Airyscan confocal images of *C. difficile* 630 cells during and immediately after cell division with HADA labelled peptidoglycan cell wall (blue) and new surface SlpA_R20291_ immunolabeled with Cy5 (white). Large, dark areas lacking HADA staining mark peptidoglycan synthesis at the septum between cells or a newly produced cell pole. Scale bar indicates 3 µm. On the right-hand side of each row is a schematic diagram illustrating the position of new surface SlpA_R20291_ (HMW-SLP, spotted light blue and LMW-SLP, spotted orange) as detected in the corresponding microscopy images against the position of endogenous surface SlpA_630_ (HMW-SLP, dark blue and LMW-SLP, red). The position of newly synthesized cell wall is displayed in brown/white stripes.

During cell division, a large amount of new surface SlpA can be detected at the septum in most dividing cells (Fig. 3). 49 cells that showed a decrease in HADA staining across the septum were chosen at random, 44 of these (89.8%) had clear Cy5 signal (new SlpA_R20291_) while 5 (10.2%) did not. This staining pattern suggests that S-layer is actively formed on the mother cell over the newly synthesized peptidoglycan preparing each daughter cell with a new S-layer cap before cell division is complete. Numerous cells also displayed areas of new peptidoglycan at one of their poles that co-insided with staining of new S-layer (Fig. 3). Of 44 randomly chosen cells with a low HADA signal at one pole, indicative of a recent cell division, 34 (77.3%) also displayed a strong polar signal for new S-layer. We interpret these as new daughter cells that have completed cell division during the HADA stain chase as detected in live cell imaging (Fig. 2a and Supplementary Movie 1). New polar S-layer can be sorted into three categories: staining distributed over the whole cell cap, on the tip of the cap or at the sides of the new cap close to the older peptidoglycan (Fig. 3). Of 141 cells displaying Cy5 signal at a low HADA stained peptidoglycan at the cell pole, 32 cells displayed whole cell cap signal, while 42 showed signal at the cell tip and 67 towards the sides of the new cap. Daughter cells with their poles completely covered in new S-layer have most likely expressed SlpA_R20291_ throughout cell division and have SlpA_R20291_ distributed all over the new S-layer cap. The apex of the cell cap marks the final place of new daughter cell formation and those caps stained just at the tip have probably expressed SlpA_R20291_ towards the final stages of division as the two daughter cells separate and the cap is completed. Areas stained at the connecting edge between the pole and the main body of the cell must represent areas of growth once the S-layer cap was fully formed when cell division was completed. Together, these staining patterns support the hypothesis that S-layer is assembled on the mother cell at the septum to form polar caps for the daughter cells to maintain a continuous protective barrier following cell separation.

### SecA2 localization

**Figure 4:**
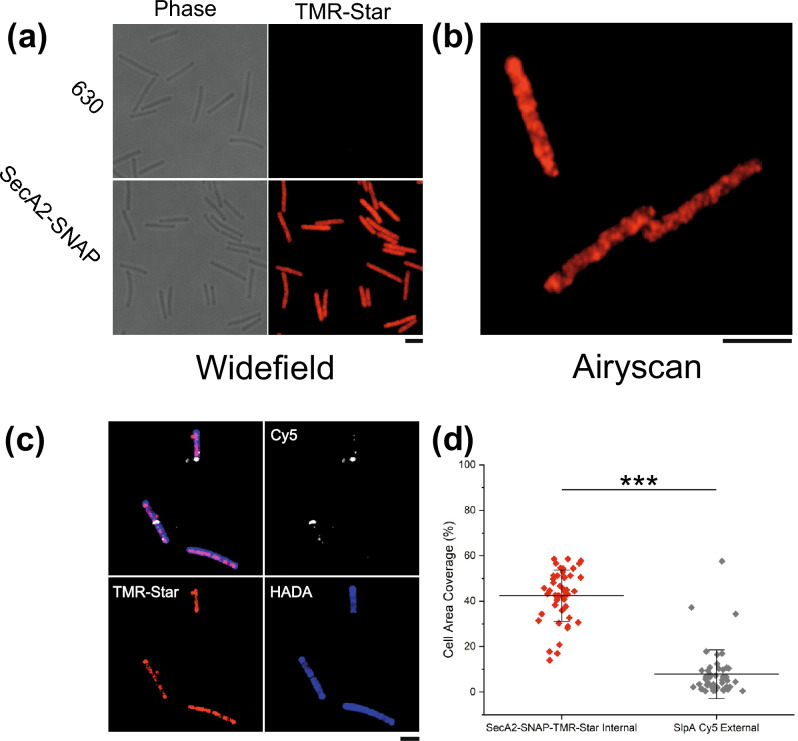
SecA2-SNAP localization and new S-layer. (**a**) Widefield phase contrast (left panels) and fluorescent (right panels) images of wild type *C. difficile* 630 or 630 *secA2-snap* cells stained with TMR-Star (red). Scale bar indicates 3 µm. (**b**) Airyscan confocal image displaying SecA2-SNAP-TMR-Star signal distribution in *C. difficile* 630 cells. Scale bar indicates 3 µm. (**c**) Airyscan confocal image showing the localization of SecA2-SNAP-TMR-Star (red) in relation to the synthesis of peptidoglycan (dark patches lacking blue HADA stain) and newly synthesized S-layer (Cy5, white). Scale bar indicates 3 µm. (**d**) Graph displaying the proportion of cell area with internal SecA2-SNAP-TMR-star signal or outline with R20291-LMW-SLP-Cy5 signal. Mean and SD of results from n = 47 cells is shown. One-way ANOVA analysis identified a significant difference in mean values *p < 0.0001.

As newly assembled S-layer is formed at specific points on the cell surface (Fig. 3) we wanted to determine if these areas correlate with concentrated points of SlpA secretion from the cytosol. Having designated sites of secretion would allow the efficient targeting of S-layer precursors to where they are needed. As it has been shown that SecA2 is essential for cell survival^13^ and performs a critical role in SlpA secretion^8^, we assumed that intracellular positioning of SecA2 will reveal where SlpA is secreted. To confirm this, we set out to create a functional, fluorescently tagged SecA2 for monitoring SecA2 localization by microscopy. A *C. difficile* strain 630 mutant was generated that encodes a C-terminal SNAP-tagged SecA2 (SecA2-SNAP) on the genome in the original locus and under the control of the native promoter. SecA2-SNAP was the only SecA2 protein detected in membrane fractions by western immunoblot analysis and, when stained with TMR-Star, this protein species was the only one visualized by in-gel fluorescence (Supplementary Fig. 4). Cells expressing SecA2-SNAP displayed similar growth dynamics to the wild-type parental strain (Supplementary Fig. 4), suggesting that the fusion protein is fully functional as SecA2 is essential for growth^13^. Imaging cells by widefield microscopy revealed that SecA2 is distributed throughout the cell and not localized to specific areas (Fig. 4a). Higher resolution, Airyscan confocal images revealed the same widespread distribution but with pockets of higher intensity signal (Fig. 4b). By combining SecA2 localization with HADA chase staining and new S-layer labelling, no correlation between SecA2 within the cell and areas of newly synthesized S-layer on the cell periphery could be identified (Fig. 4c). To confirm this quantitatively we performed signal distribution analysis around the cell boundary for Cy5 (new SlpA_R20291_ exposed on the cell surface) and cell volume for TMR-Star (SecA2). Signal was detected on average covering 42% of the apparent cell volume for SecA2 but only 14% of the cell boundary for new SlpA (Fig. 4d). Together these data suggest that SecA2 localization, and hence the localization of SlpA secretion, is not the determining factor in localization of new S-layer growth at the surface.

### SlpA secretion

**Figure 5:**
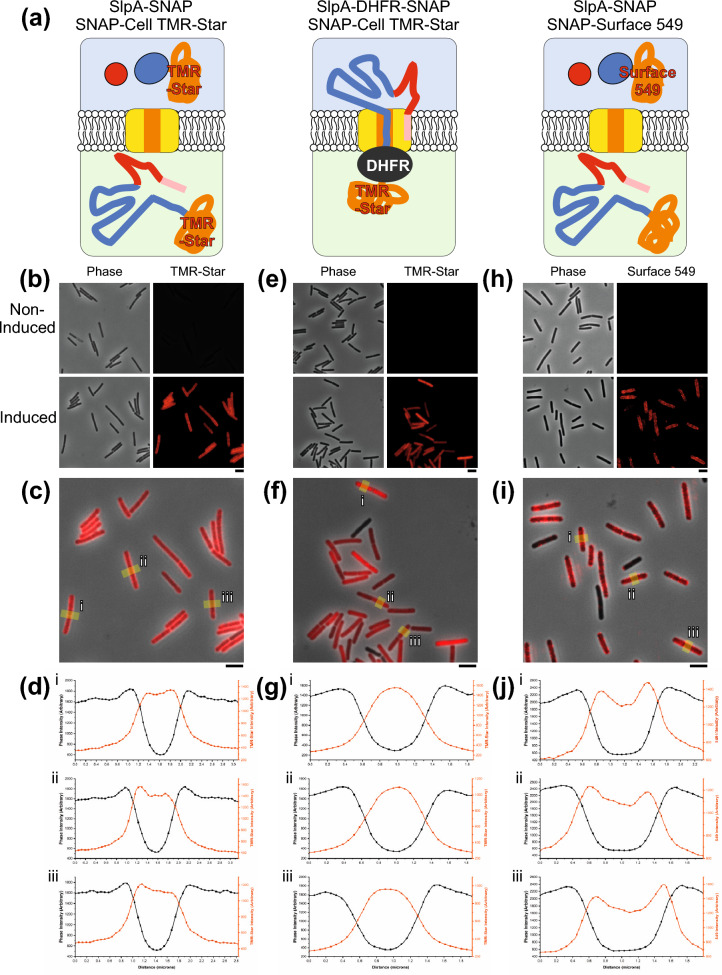
Sites of S-layer secretion. (**a**) Schematic diagram illustrating the position of stained SNAP tagged SlpA constructs expressed in *C. difficile* 630 cells: SNAP-Cell TMR-Star stained SlpA-SNAP (left panel) or SlpA-DHFR-SNAP (center panel) and SNAP-Surface 549 stained SlpA-SNAP (right panel). Colored as in Fig. 1b with SNAP tags represented as an orange coil. SlpA-SNAP is exported and cleaved into LMW-SLP and HMW-SLP-SNAP. The DHFR domain (dark gray oval) of SlpA-DHFR-SNAP blocks the translocon channel during export, leaving the TMR-Star bound SNAP tag in the cytosol. SNAP-Surface 549 stains extracellular HMW-SLP-SNAP only. (**b**) Widefield phase contrast (left panels) and fluorescent SNAP-Cell TMR-Star signal (right panels) of *C. difficile* 630 cells stained with SNAP-Cell TMR-Star imaged with and without induction of SlpA-SNAP expression. Scale bar indicates 3 µm. (**c**) Overlay of fluorescent signal in the induced sample (from (**b**)) with areas taken for the plot profiles labelled (yellow lines, i–iii). Scale bar indicates 3 µm. (**d**) SlpA-SNAP-Cell TMR-Star profile plots of i-iii (from (**c**)) of phase contrast signal (black) and SNAP-Cell TMR-Star signal (red). (**e**) SlpA-DHFR-SNAP in *C. difficile* 630 cells (labelled as in (**b**)). (**f**) Overlay of signal in the induced sample (from (**e**), labelled as in (**c**)). (**g**) SlpA-DHFR-SNAP-Cell TMR-Star profile plots of i–iii (from (**f**)) (labelled as in (**d**)). (**h**): Widefield phase contrast (left panels) and fluorescent SNAP-Surface 549 signal (right panels) of *C. difficile* 630 cells stained with SNAP-Surface 549 imaged with and without induction of SlpA-SNAP expression. (**i**) Overlay of signal in the induced sample (from (**h**), labelled as in (**c**)). (**j**) HMW-SLP-SNAP-Surface 549 profile plots of i-iii (from (**i**)) of phase contrast signal (black) and SNAP-Surface 549 (red).

Although SecA2 was visualized throughout the cell, SecA2 has additional secretory substrates^8^ so it is possible that secretion of SlpA itself may be localized. In the experiments described above we used immunofluorescence to detect where new SlpA subunits were inserted into the S-layer. As the S-layer can exclude proteins as small as lysozyme^3^, immunofluorescence does not allow visualization of intracellular or extracellular SlpA present below the S-layer. To visualize these potential pools of protein and determine where SlpA is being secreted, two different SlpA fusion proteins were constructed, an SlpA_630_-SNAP fusion that can be secreted and is found in the extracellular fraction and a SNAP tagged SlpA_630_-dihydrofolate reductase (SlpA_630_-DHFR-SNAP) fusion that associates with the cellular fraction (Fig. 5a and Supplementary Fig. 5). DHFR is a fast folding protein that has been used to block and probe protein translocation mechanisms for many years^14–17^. Expressing SlpA_630_-DHFR-SNAP decreases the secretion of native *C. difficile* extracellular proteins (Supplementary Fig. 5) and leads to the build-up of precursor SlpA within the cell (Supplementary Fig. 6), consistent with DHFR blocking SecA2-mediated translocation. This effect requires the SlpA signal sequence, as an SlpA_630_-DHFR-SNAP lacking the N-terminal signal sequence no longer blocks protein translocation (Supplementary Fig. 5). Together these findings show that the SlpA_630_-DHFR fusion used here is specifically targeted to and occupies the same secretory channel required for wild-type SlpA secretion. To prevent protein secretion, the DHFR domain must first fold correctly^15^ and will therefore only prevent secretion of a fusion protein that is targeted to the post-translational pathway. The lack of detectable SlpA_630_-DHFR-SNAP in the extracellular fraction (Supplementary Fig. 5) shows for the first time that SlpA is exclusively post-translationally translocated. Using these two SNAP fusion proteins we can now probe the intercellular localization of SlpA secretion (SlpA_630_-DHFR-SNAP) and localization once secreted (SlpA_630_-SNAP).

**Figure 6:**
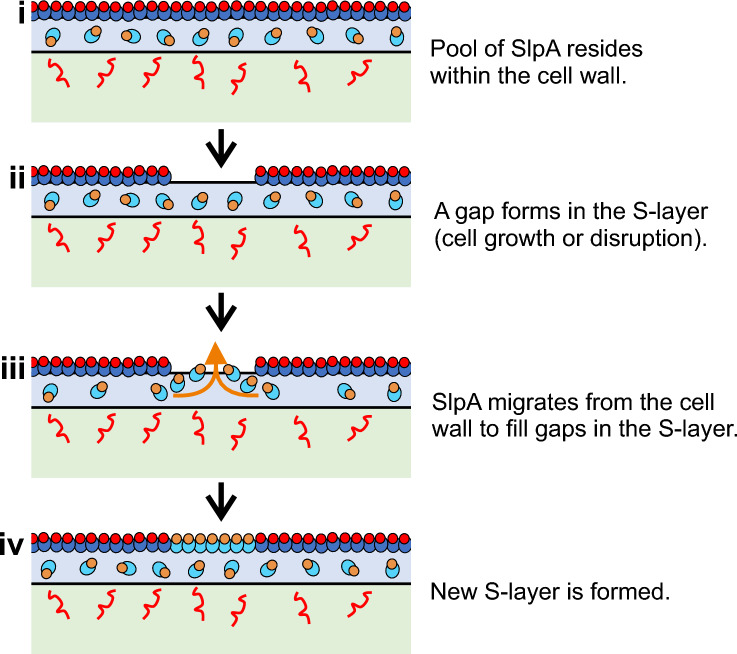
Model of S-layer in the cell envelope. Schematic flow diagram of SlpA secretion and S-layer formation (colored as in Fig. 1c). During normal cell growth SlpA is targeted by SecA2 for secretion all over the cytosolic membrane. A store of SlpA resides within the cell wall where it is processed ready for integration into the S-layer (**i**). Gaps may form in the S-layer due to cell growth or injury (**ii**). SlpA in the cell wall diffuses out (**iii**) and fills openings in the S-layer (**iv**).

After secretion, the SlpA_630_-SNAP protein is processed as normal by the cell surface localized cysteine protease Cwp84 (Fig. 1b), yielding the LMW-SLP subunit and a SNAP tagged HMW-SLP (HMW-SLP-SNAP)(Supplementary Fig. 5a). Using a cell permeable dye, TMR-Star, we observed a diffuse distribution of HMW-SLP-SNAP throughout the cell (Fig. 5b,c). Signal intensity analysis of 69 stained cells revealed a broad cross-section of TMR-Star intensity across the cell width (Phase vs TMR-Star signal, Fig. 5d), with smaller peaks in TMR-Star intensity which correlate with the cell periphery seen in 38% of cells (Fig. 5d and Supplementary Table S2). This is consistent with the detection of HMW-SLP-SNAP TMR-Star in the extracellular fraction (Supplementary Fig. 5). Cells expressing SlpA_630_-DHFR-SNAP show some heterogeneity of expression (Fig. 5e,f), perhaps caused by leaky transcription leading to a negative selective pressure for the plasmid due to the drastic effects this DHFR fusion protein has on secretion (Supplementary Fig. 5). Again, the signal from SlpA_630_-DHFR-SNAP appears diffuse throughout the cell and not located at specific sites (Fig. 5e,f). Signal intensity analysis of 44 cells revealed a narrow TMR-Star signal peak towards the interior of 93% of cells where the phase signal is low (Fig. 5g and Supplementary Table S2), suggesting a more intracellular location for SlpA_630_-DHFR-SNAP than HMW-SLP-SNAP which is consistent with SlpA-DHFR-SNAP being trapped in the cell at the membrane (Supplementary Fig. 5).

To obtain a clearer image of newly secreted extracellular HMW-SLP-SNAP, cells were treated with SNAP-Surface 549 (Fig. 5h,i), a dye that is not cell permeable and that specifically stains extracellular proteins (Supplementary Fig. 5). This dye will reduce the intracellular SlpA_630_-SNAP background staining observed with SNAP-Cell TMR-Star, while efficiently staining the extracellular pool of processed HMW-SLP-SNAP (Fig. 5a and Supplementary Fig. 5). Widefield imaging of cells stained with SNAP-Surface 549 revealed a patchy distribution that appeared surface localized (Fig. 5h,i), confirmed by signal intensity analysis across a transverse section of 42 stained cells that showed 79% of cells with signal centered around the cell boundary (two peaks, Fig. 5j and Supplementary Table S2). Given the apparent patchy distribution we then analyzed SNAP-Surface 549 stained cells by Airyscan confocal microscopy (Supplementary Fig. 7). This showed an uneven punctate distribution of HMW-SLP-SNAP all over the cell.

As the distribution of SecA2 (Fig. 4b) and HMW-SLP-SNAP (Supplementary Fig. 7) did not agree with our observation of new S-layer assembly at discrete points on the cell surface (Figs. 2, 3 and 4), we sought to reconcile this by simultaneously visualizing both the total extracellular pool of secreted SlpA and that portion which is surface exposed within the S-layer. SlpA_630_-SNAP was expressed in strain R20291 to allow differentiation from the native SlpA_R20291_ by immunofluorescence. Cells were stained with SNAP-Surface 549, to label the total extracellular pool, and with an antibody specifically recognizing the 630 LMW-SLP subunit to label the subset that was surface exposed in the S-layer (Supplementary Fig. 8). As seen before, SNAP-Surface 549 signal was detected all around the cell while antibody labelling only revealed S-layer exposed protein at discrete loci on the cell surface. To quantify this we performed signal intensity analysis around the cell boundary, revealing an average of 80% coverage for SNAP-Surface 549 signal compared with only 15% coverage for new SlpA exposed on the cell surface.

Taken together these data suggest that SlpA secretion occurs over the majority of the cell surface and not just at sites where new S-layer is being formed, with insertion into the existing S-layer constrained to sites of S-layer breakage due to cell growth. To confirm this we expressed SlpA_630_ in a strain lacking an pre-existing S-layer^3^ (Supplementary Fig. 9). With a short 5 min expression we could detect new SlpA over a large portion of the cell surface, in a pattern similar to that seen for the localization of SecA2-SNAP (Fig. 4), SlpA_630_-SNAP and SlpA_630_-DHFR-SNAP (Fig. 5).

## Discussion

For an S-layer to function correctly it must completely encapsulate the cell^3,18^. We propose here that S-layer is assembled at areas of newly synthesized peptidoglycan to maintain a stable S-layer that continually protects the *C. difficile* cell. Although newly synthesized SlpA is secreted from all regions of the cell, only a relatively small proportion of this was detected at the surface. This irregularity suggests that *C. difficile* possess reserves of SlpA beneath the S-layer in the cell wall (Fig. 6). This is consistent with previous observations suggesting the presence of an S-layer protein pool in the *Bacillus stearothermophilus* cell wall^19^. We propose that multiple points of interaction between the S-layer and PS II are not permissive for lateral S-layer movement on the cell surface and, as a result, growth of the underlying peptidoglycan sacculus produces fissures in the S-layer that are then filled using SlpA subunits present in the interwall pool. Although excess SlpA production and storage will be quite energetically expensive for the cell, this reservoir of SlpA could provide a positive fitness advantage by allowing cells to respond quickly to repair gaps in this critical barrier (Fig. 6). Examples of self-repair mechanisms are present thorough all forms of life from intracellular vesicular mediated membrane healing^20,21^ up to a tissue level such as wound healing^22^. In addition to providing a buffer to reduce the amount of de novo SlpA translation and translocation required to safely complete cell division, this stockpile of SlpA in the cell wall may also allow replacement of individual S-layer subunits lost due to damage. However, the methods employed here lack the sensitivity that would be required to visualize these single molecule events.

Although S-layers are commonly considered as continuous 2D crystals covering the cell surface^4^, fractures in the S-layer must form to allow the cells to grow. Our data suggests that these fractures coincide with new peptidoglycan synthesis and that new SlpA emerges through these gaps to be incorporated into the crystalline lattice. Higher resolution imaging techniques may allow the direct observation these gaps in the S-layer and how the separate S-layer sections assemble. When new S-layer and secreted HMW-SLP-SNAP was labelled (Fig. 2b, Supplementary Figs. 3 and 7) and intracellular SecA2-SNAP was detected (Fig. 4b), regular patterns and sometimes diagonal staining could be seen along the longitudinal axis of the cell. These patters may relate the localization of SecA2 and newly forming S-layer in line with intracellular cytoskeletal and motor proteins that power cell growth^23^. It was recently demonstrated that in *C. crescentus*, new S-layer growth is localized to both poles in addition to areas of cell wall growth^24^. Here we have shown that new S-layer in *C. difficile* is usually limited to one pole, correlating with peptidoglycan synthesis. Unlike in *C. crescentus*, we observed that new *C. difficile* S-layer was incorporated mainly at the septum during cell division, rather than at the pole during cell growth. Comerci et al., suggest that crystalline patches at areas of such curvature contain more defects, allowing more local SLP incorporation. As we do not see equivalent incorporation of new S-layer at the *C. difficile* poles, we suggest that this S-layer may be more amenable to surface curvature.

We have also demonstrated for the first time that SlpA is secreted post-translationally. Proteins transported in this way usually interact with cytosolic chaperones that prevent folding prior to translocation^25^. The identity of these chaperones and the exact role SecA2 plays in SlpA secretion has yet to be determined. Since SlpA must undergo a post-secretion protease modification (Fig. 1)^5^, having a dwell time in the cell wall will allow time for correct processing. However this also poses the question of how S-layer components located there are prevented from oligomerizing prior to assembly at the surface^26^. It is tempting to speculate that the S-layer assembly pathway may also involve extracellular chaperones to facilitate processing and targeting while preventing premature self-assembly. The revelation that there is a pool of SlpA in the cell wall and the accessibility of the cell wall to drugs may provide opportunities for the identification of novel narrow spectrum targets that affect the assembly or processing of this essential virulence factor.

In summary, we have found that new S-layer is formed at sites of peptidoglycan synthesis and there is an underlying supply of the S-layer precursor, SlpA, located throughout the cell wall.

## Methods

### Media and growth conditions

All strains, plasmids and oligonucleotides used in this investigation are displayed in Supplementary Table S3. CA434 and NEB5α *E. coli* were grown in LB broth or on LB agar supplemented when required with 15 µg/ml chloramphenicol for plasmid selection. *C. difficile* were grown in reduced TY (3% tryptose, 2% yeast extract) broth or on Brain Heart Infusion agar under strict anaerobic conditions. Cultures were supplemented with 15 µg/ml thiamphenicol when selecting for plasmids.

For SlpA-hDHFR-Strep Tag II expression, bacteria were subcultured from overnight cultures to an OD_600nm_ of 0.05, grown for 30 min and then supplemented with 200 µM methotrexate. Bacteria were then grown for a further 30 min before expression was induced with 20 ng/ml anhydrotetracycline (Atc). Bacteria were grown for a further 3 h before harvesting at 4,000×*g*, for 10 min at 4 °C.

### Molecular biology

Chemically competent *E. coli* were transformed by heat shock using standard methods and plasmids were transferred to *C. difficile* strain 630 by conjugation using the *E. coli* donor strain CA434^27^. Standard techniques were used for PCR, restriction digestion, ligation and Gibson assembly. DNA modifications were performed using Phusion High-Fidelity DNA Polymerase (Thermo Fisher) and Q5 Site-Directed Mutagenesis Kit (NEB) as per manufacturers’ instructions.

### Plasmid construction

pRFP233^3^ was modified to add the tetracysteine (Tc) tag encoding sequence into *slpA* such that a modified LMW-SLP is produced with FLNCCPGCCMEP added to a surface-exposed loop. The plasmid was linearized by inverse PCR using oligonucleotides RF411 and RF412, deleting 150 bp of the LMW-SLP coding sequence. A synthetic DNA fragment including the deleted 150 bp and 36 bp encoding the Tc tag was inserted by Gibson assembly, yielding plasmid pRPF238.

For the addition of the SNAP tag to SecA2, *secA2* was amplified using RF216 and RF217 from gDNA and digested using SacI/XhoI. *snap* was amplified from pFT46^28^ using RF218 and RF219 and digested with BamHI/XhoI. These fragments were then ligated into SacI/BamHI digested pRPF144^8^ in a 3-fragment ligation reaction yielding pJAK014. *secA2*-*snap* was excised using SacI/BamHI and ligated into similarly treated pRPF185 yielding pJAK038.

Modification of the *C. difficile* 630 genome was achieved by allele exchange as described previously^29^. The *snap* coding sequence and the last 1.2 kb of *secA2* was amplified by PCR using RF635 and RF636, using pJAK014 as a template. 1.2 kb downstream of *secA2* was amplified by PCR using RF637 and RF638 using gDNA as a template. pMTL-SC7315^29^ was linearised by PCR using RF311 and RF312. The three fragments were ligated by Gibson assembly yielding pJAK067. To improve the enzymatic activity of the expressed fusion protein, the size of the linker between SecA2 and SNAP was increased. pPOE032 was prepared by inverse PCR of pJAK067 with RF1079 and RF1080 via a site-Directed Mutagenesis Kit (NEB) as per manufacturer’s instructions.

An *slpA*_*630*_*-strep tag II* encoding SacI/BamHI insert was excised from pRPF173 and ligated into a SacI/BamHI digested pRPF185 yielding pPOE005. pPOE005 was modified to add the coding sequence for *hDHFR-myc* within *slpA*_*630*_*-strep tag II*. *hDHFR-myc* was amplified using RF721 and RF722 and ligated into XhoI linearized pPOE005 to give pPOE002. Sequence encoding a 3xHA tag was added to *slpA*_*630*_*-hDHFR-myc* by inverse PCR of pPOE005 with RF811 and RF812 by site-directed mutagenesis, as described earlier, to create pPOE003. The sequence encoding the SlpA_630-_hDHFR-Strep Tag II signal peptide in pPOE002 was removed by inverse PCR site-directed mutagenesis with RF789 and RF790 to create pPOE011. pPOE023 was prepared by ligation of the *slpA*_*630*_ SacI/XhoI insert from pPOE005 into SacI/XhoI digested pJAK038 vector.

To add a SNAP tag to SlpA_630_-hDHFR-Strep Tag II, pPOE002 was linearized by PCR using RF866 and RF867. *snap* was amplified from pJAK038 using RF868 and RF869. These fragments were then NotI/BamHI digested and ligated yielding pJAK085.

### Microscopy

SNAP Cell TMR-Star and SNAP-Surface 549 Staining: cells were grown from an OD_600nm_ 0.05 to 0.4 and treated with 250 µM TMR-Star for at least 30 min. Transient expression of SlpA-SNAP or SlpA-DHFR-SNAP was induced for 10 min with 10 ng/ml Atc before fixation or 20 ng/ml for 1 h for in-gel fluorescence experiments.

HADA Staining: Cells were grown to an OD_600nm_ of approximately 0.1 before the addition of 0.5 mM HADA and continued growth for at least 2 h to an OD_600nm_ of 0.5–0.6. To chase the HADA staining, cells were harvested at 4,000×*g* for 5 min, washed once by resuspension in 8 ml reduced TY and finally resuspended in 2 × the original volume of reduced TY media before continuing growth for up to 30 min. In the case of transient SlpA_R20291_ expression the cells were grown for 25 min before inducing the expression with 100 ng/ml Atc for the final 5 min before fixation.

Live-cell sample preparation: After the wash with 8 ml TY (as described above), HADA stained cells were resuspended in reduced TY to an OD of approx. 50. 0.5 OD600U of cells were transferred in an anaerobic chamber to a glass bottom petri dish (ibidi) at the interface between the glass coverslip and a 1% agarose pad that covered the entire surface of the dish and had been reduced for at least 3 h. The petri dish was tightly wrapped in parafilm under anaerobic conditions before immediately been transferred at 37 °C to a widefield microscope chamber pre-heated to 37 °C for imaging.

Fixation: Cells were harvested at 4,000×*g* for 5 min at 4 °C, washed two times in 1 ml ice cold PBS with spins at 8,000×*g* for 2 min at 4 °C before being fixed with 4% paraformaldehyde in PBS for 30 min at room temperature. After fixation, cells were washed three times in PBS. For immunofluorescence the fixed cells were blocked overnight with 3% BSA in PBS at 4 °C. Cells were harvested at 8,000×*g* for 2 min at 4 °C, resuspended in 1:500 Primary antibody (Mouse Anti-027 SlpA_R20291_ LMW-SLP) and incubated at room temperature for 1 h. Cells were then washed three times in 1 ml 3% BSA in PBS before being resuspended in 1:500 secondary antibody (Goat anti-mouse-Cy5, Thermo Fisher). Cells were incubated for 1 h at room temperature then washed three times in 3% BSA in PBS before being resuspended in PBS. Washed cells were dried down to glass cover slips and mounted with SlowFade Diamond (Thermo Fisher).

Images were taken on a Nikon Ti eclipse widefield imaging microscope using NIS elements software or a ZEISS LSM 880 with Airyscan using ZEN imaging software. Image J based FiJi was used for image analysis. Cell signal coverage was performed in Fiji firstly by creating a cell mask from phase contrast or HADA staining. To remove large aggregates, cells were filtered by size and those on image borders were excluded. When looking at florescent signal on the cell surface, outlines of the cell masks were drawn, and image calculations used to isolate signal at the cell periphery. Bespoke macros were then used to calculate the total possible area around the cell and the proportion of this area which was covered in significant florescent signal.

### Cell fractionation

Extracellular protein extraction: Cells were harvested at 4,000×*g* for 5 min at 4 °C. In all the following wash steps bacterial cells we centrifuged at 8,000×*g* for 2 min. Pellets were washed twice by resuspension in 1 ml ice cold PBS. Cells were treated with 10 µl per OD_600nm_U of extraction buffer (0.2 M Glycine, pH 2.2) and incubated at room temperature for 30 min to strip extracellular proteins. Stripped cells were harvested and the supernatant, containing extracellular protein, was taken and neutralized with 0.15 µl 1.5 M Tris pH 8.8 per 1 µl extract. The stripped cells were washed twice in 1 ml ice cold PBS before being frozen at -80C. Cells were thawed and then resuspended in 11.5 µl per OD_600nm_U cell lysis buffer (PBS, 1 × protease inhibitor cocktail, 5 mM EDTA, 20 ng/ml DNase, 120 mg/ml purified CD27L endolysin^30^). Lysis was induced by incubating at 37 °C shaking for 30 min. Cell membranes were harvested by centrifugation at 20,000×*g* for 20 min and the soluble intracellular protein fraction retained before the pellet was washed twice with 1 ml PBS. Membranes were solubilized using 11.5 µl per OD_600nm_U solubilization buffer (1 × PBS, 1 × protease inhibitor cocktail, 5 mM EDTA, 20 ng/ml DNase, 1.5% sarkosyl) and agitated by rotating for 1 h at room temperature. Insoluble material was harvested at 20,000×*g* for 5 min and the solubilized membrane fraction was taken. Alternatively, for the SNAP tagged SlpA constructs; cells lysates were supplemented with 1.5% sarkosyl, incubated for 1 h and harvested at 20,000×*g* for 5 min to create a total cellular extract.

### Protein gels

Proteins were separated using standard SDS-PAGE techniques on a mini-protein III system (Bio-Rad) before being either; analyzed for in-gel florescence on a ChemiDoc imaging system (Bio-Rad), stained with Coomassie or transferred to nitrocellulose membranes using a semi-dry blotter (Bio-Rad) for western blot analysis. Membranes were probed with either a rabbit anti-SecA2 (raised against SecA2-Strep-tag II expressed in *E. coli*^8^), mouse anti-LMW-SLP_R20291_ (raised against LMW-SLP_R20291_-His expressed in *E. coli*), mouse anti-HA (Sigma, H9658), mouse anti-Strep-tag II (Sigma, 71590-M) or mouse anti-AtpB (VWR, AS05 085). Primary antibodies were detected with the corresponding HRP‐conjugated secondary antibody. Band intensities were measured using Image Lab Software (Bio-Rad).

### Statistics

Linear regression of HADA and Cy5 signal was performed in Microsoft office 365 Excel. To determine statistical differences in populations Origin one-way analysis of variance (ANOVA) was used, a difference with p ≤ 0.05 was considered significant.

## Supplementary information

Supplementary Information.

Supplementary Movie.

## Data Availability

All materials, data and protocols are available from the corresponding authors on request.
