# Supplementary material for: Spatial organization of Clostridium difficile S-layer biogenesis
Source: Sci Rep. 2020 Aug 24;10:14089. doi: 10.1038/s41598-020-71059-x (PMC7445750; doi:10.1038/s41598-020-71059-x)
Supplement: Supplementary file 1 — Supplementary Information. [file 41598_2020_71059_MOESM1_ESM.docx]

# Supplemental Information for:

# Spatial organization of *Clostridium difficile* S-layer biogenesis

Peter Oatley, Joseph A. Kirk, Shuwen Ma, Simon Jones, Robert P. Fagan


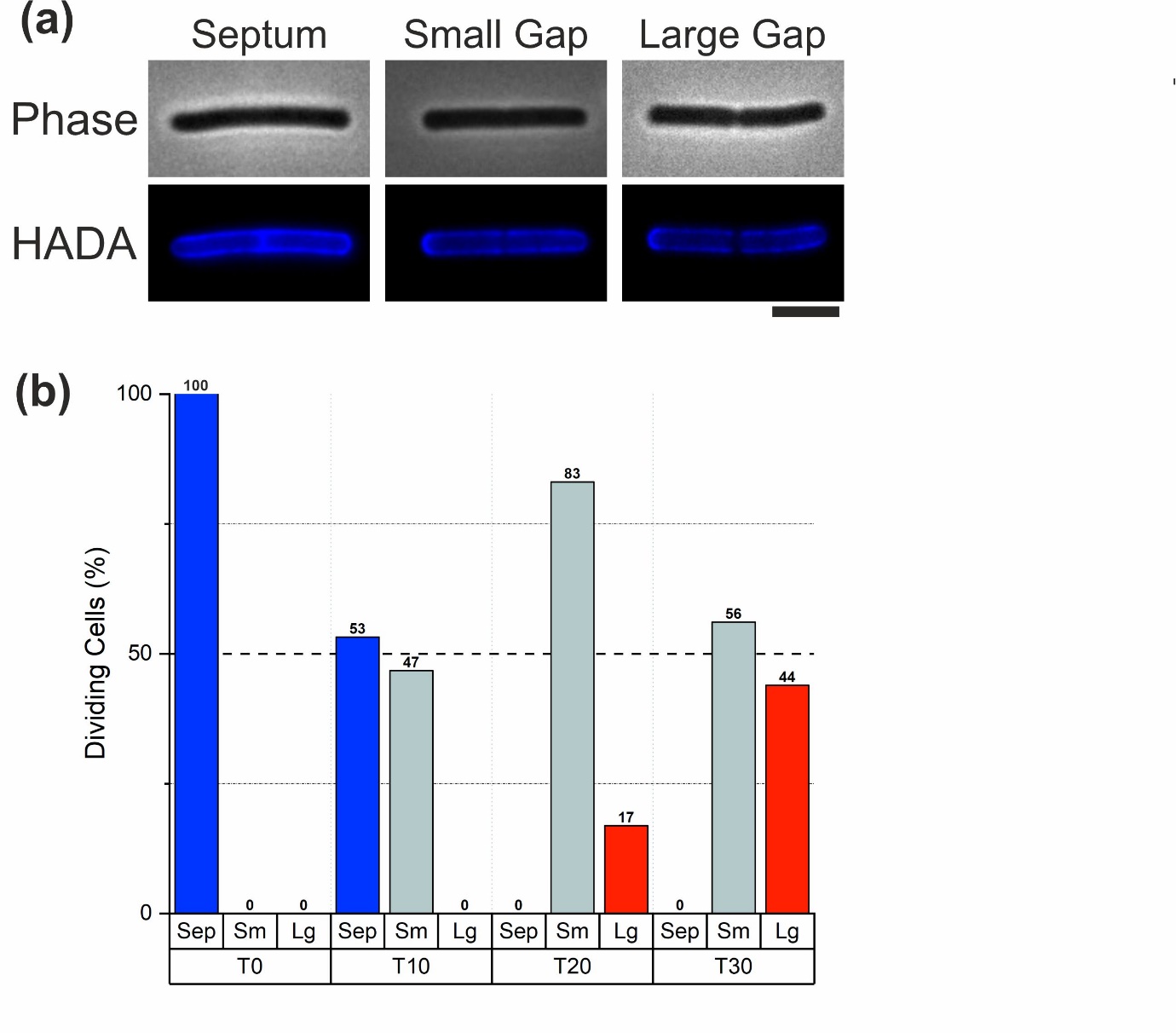


#### Supplementary Figure 1: *C. difficile* 630 HADA staining chase time course

**(a)** Widefield microscopy displaying phase contrast (upper panels) and fluorescence (lower panels) of HADA stained *C. difficile* 630 cells chased for 0, 10, 20 or 30 min without HADA (as described in Methods). HADA staining at the center of a dividing cell can be characterized as: septum stained (left) or patches of reduced HADA staining being smaller than 360 nm in length (middle) or larger (right). Scale bar indicates 3 µm.

**(b)** Graph displaying the population distribution of dividing *C. difficile* 630 cells characterized for HADA staining in widefield microscopy (as in (a)) when chased for HADA for 0, 10, 20 or 30 minutes (T0, n=56; T10, n=62; T20, n=71; T30, n=107). The percentage of the total counted population are displayed above each bar.


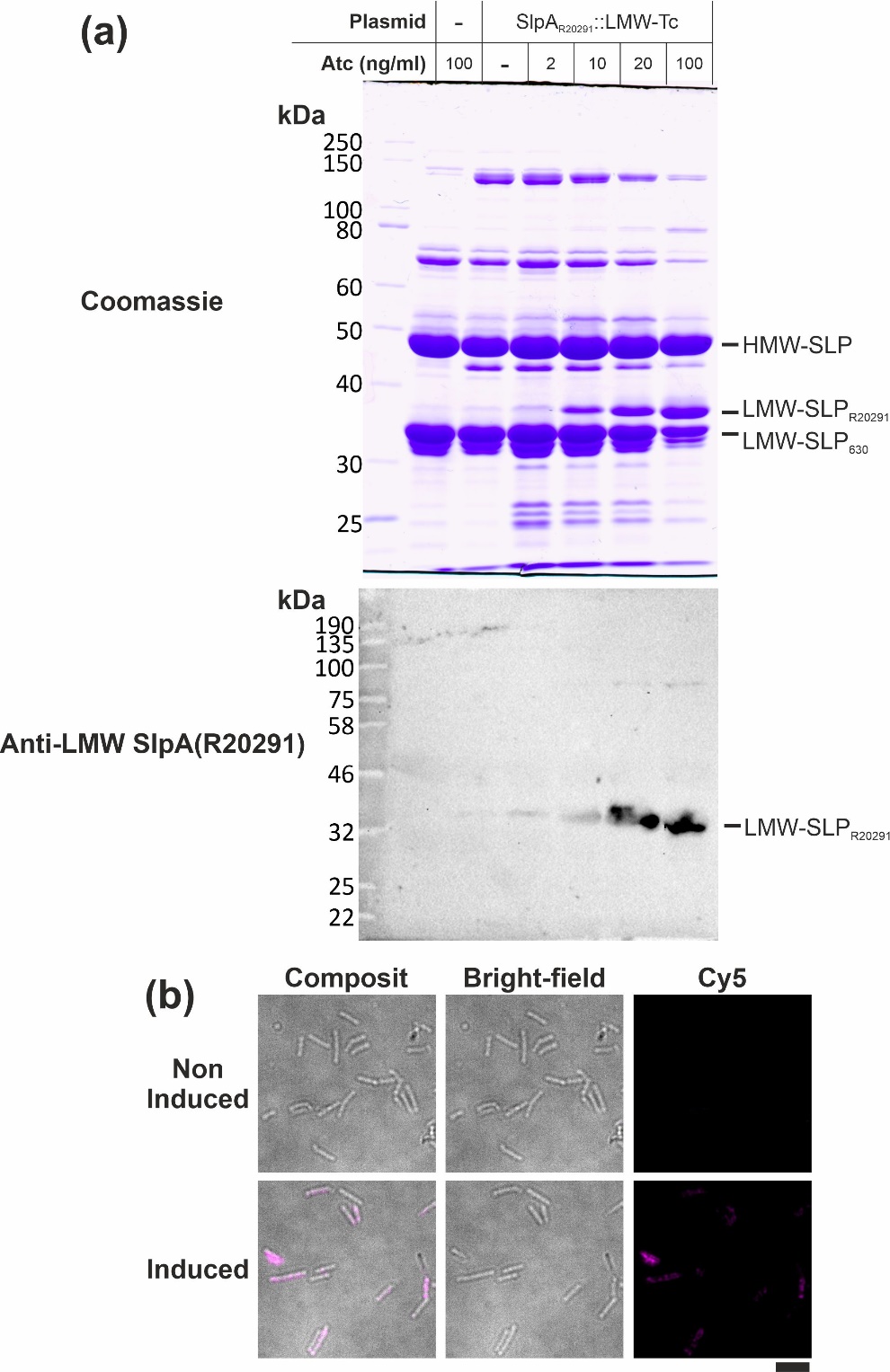


#### Supplementary Figure 2: Antibody specificity for SlpA_R20291_ LMW-SLP.

**(a)** Top panel: Coomassie stain of SDS-PAGE separated extracellular extracts from *C. difficile* 630 cells grown for three hours with the indicated amount of anhydrotetracycline (Atc) to induce protein expression. Lower panel: Western immunoblot to detect SlpA_R20291_ LMW-SLP in the same extracellular extracts.

**(b)** Widefield microscopy of *C. difficile* 630 cells with pRPF238 (encoding SlpA_R20291_::LMW- Tetracysteine. The tetracysteine tag was used during other labelling experiments that were unsuccessful (data not shown)) induced (bottom panels) or not induced (top panels) with 100 ng/ml Atc for 5 minutes. Surface SlpA_R20291_ was immunolabeled with Cy5 (magenta). Scale bar indicates 6 µm.


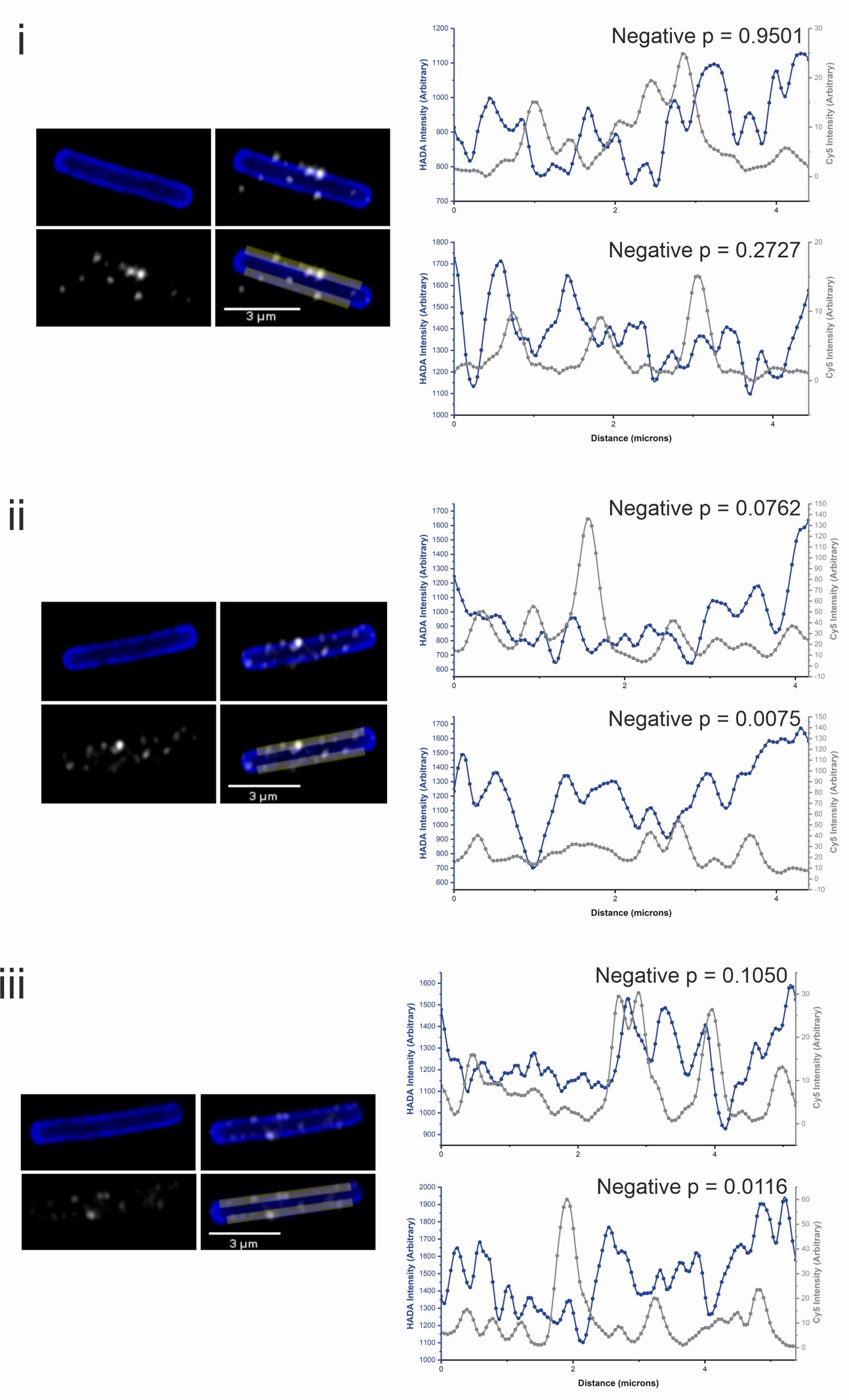


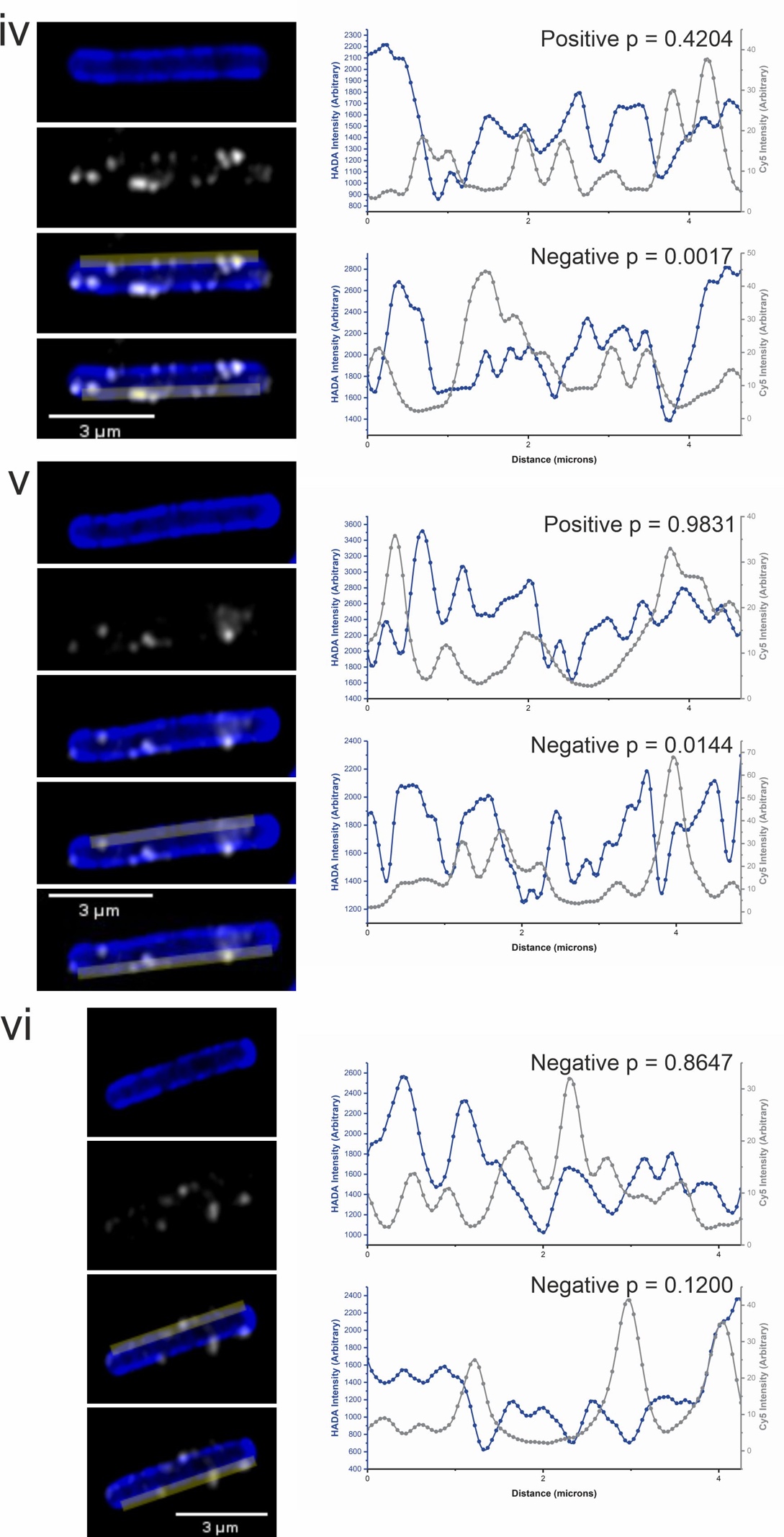


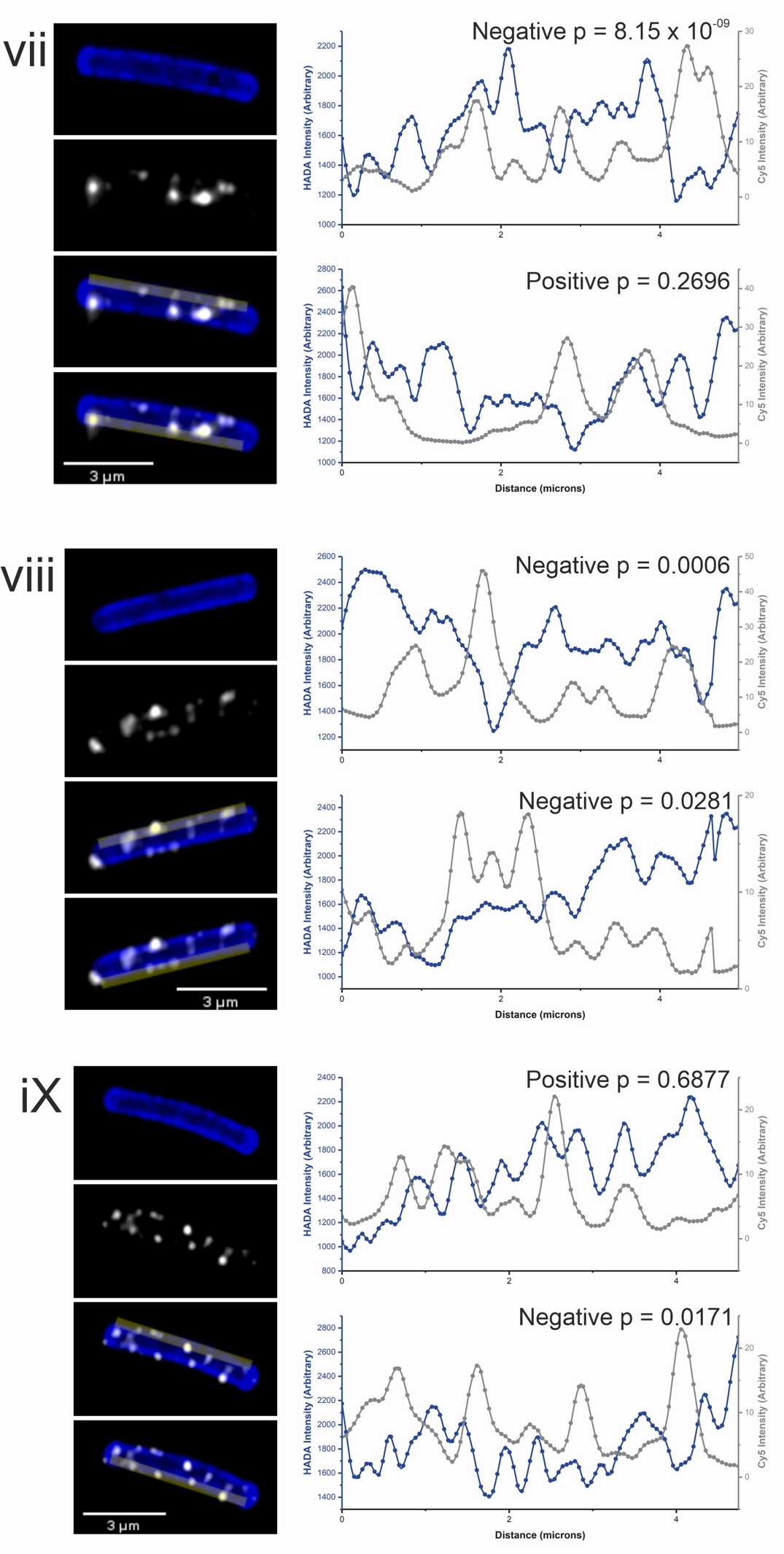


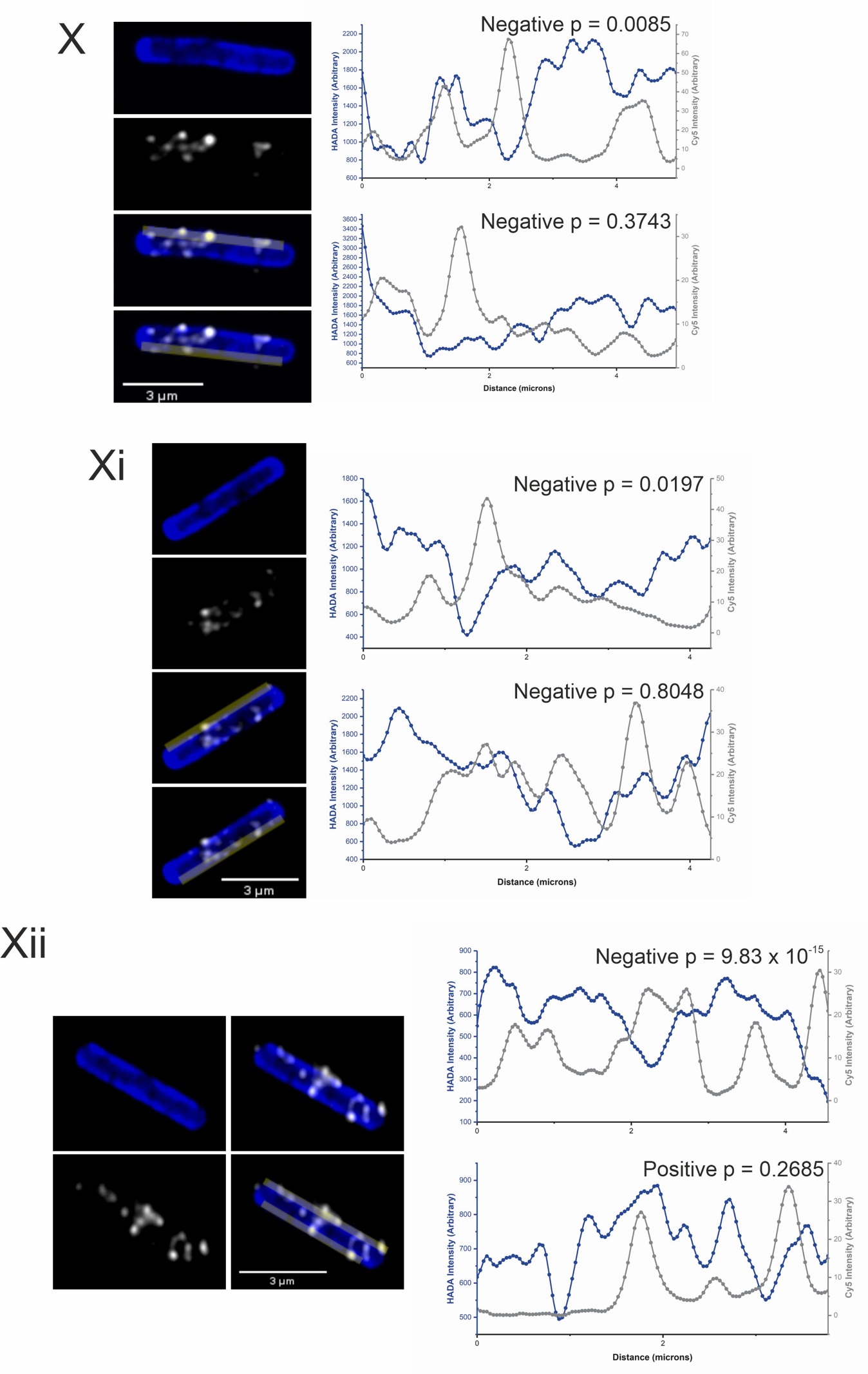


#### Supplementary Figure 3: Further images of new surface S-layer

Representative examples of Airyscan confocal images displaying *C. difficile* 630 cells prepared as in Fig. 2 with HADA label peptidoglycan cell wall (blue), new SlpA_R20291_ immunolabeled with Cy5 (white) and yellow bar regions used for intensity plot graphs. Intensity plot graphs display HADA (blue) and Cy5 (grey) signal with upper and lower graphs corresponding to the higher and lower cell region marked with yellow bars in the left panels, respectively.


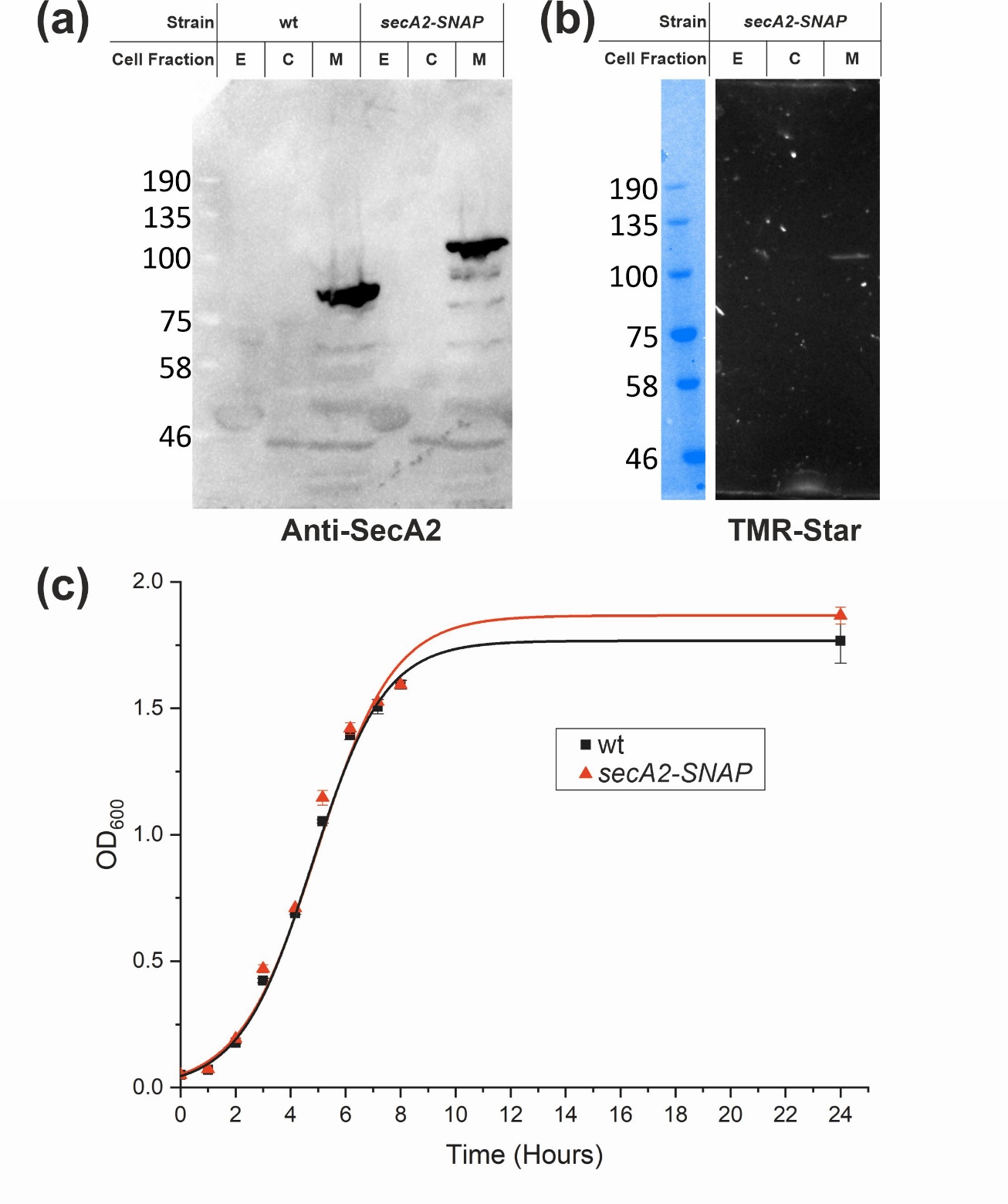


#### Supplementary Figure 4: SecA2-SNAP is functional in *C. difficile.*

**(a)** Western immunoblot showing the distribution of SecA2 in extracellular (E), cytosolic (C) and membrane (M) fractions from wild-type *C. difficile* 630 or cells expressing a genomic copy of a *secA2-SNAP* fusion, loaded at the same OD_600_U.

**(b)** In-gel fluorescence of SecA2-SNAP-TMR-Star from cell extracts expressing SecA2-SNAP (labelled as in (a)).

**(c)** Growth curves of wild-type *C. difficile* 630 (wt) or 630 *secA2-SNAP.* Following inoculation at an OD_600_ of 0.05, growth was followed by measuring OD_600_ hourly. Shown are the mean and standard error of triplcate cultures.


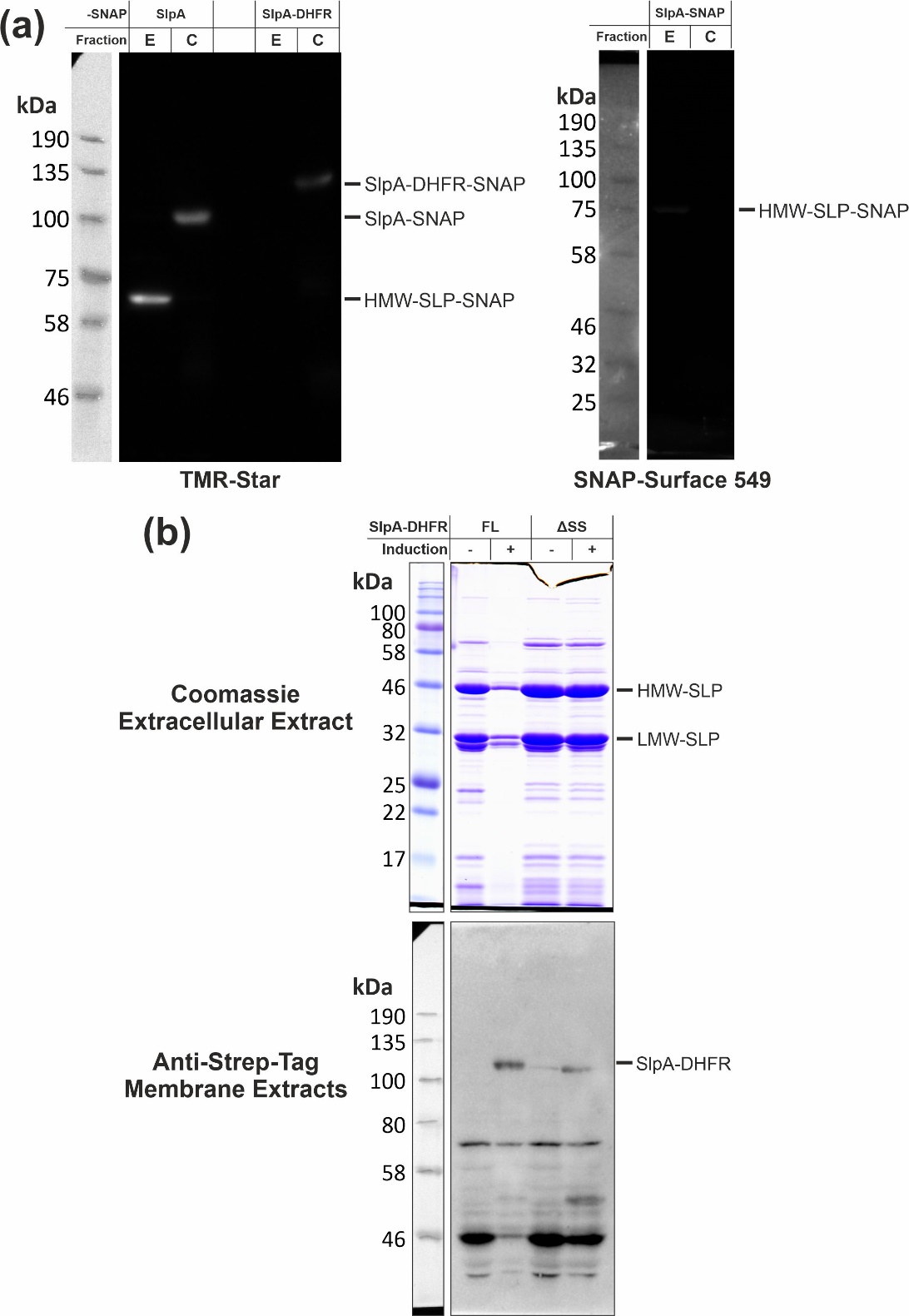


#### Supplementary Figure 5: SlpA-SNAP, SlpA-DHFR-SNAP, SlpA-DHFR expression and localization

**(a)** Fluorescence displaying SNAP-TMR-Star signal (left) or SNAP-Surface 549 (right) from extracellular (E) or cellular (C) *C. difficile* 630 extracts expressing SlpA_630_-SNAP or SlpA_630_-DHFR-SNAP, separated by SDS PAGE.

**(b)** SDS PAGE analysis of extracellular extracts stained with coomassie (upper panel) or membrane fractions analyzed by Western immunoblot with an anti-strep-tag antibody (lower panel) from *C. difficile* 630 cells expressing strep tagged full length SlpA_630_-DHFR or SlpA_630_-DHFR lacking a signal sequence (ΔSS). Protein expression was induced with 20 ng/ml Atc for 180 min as indicated.


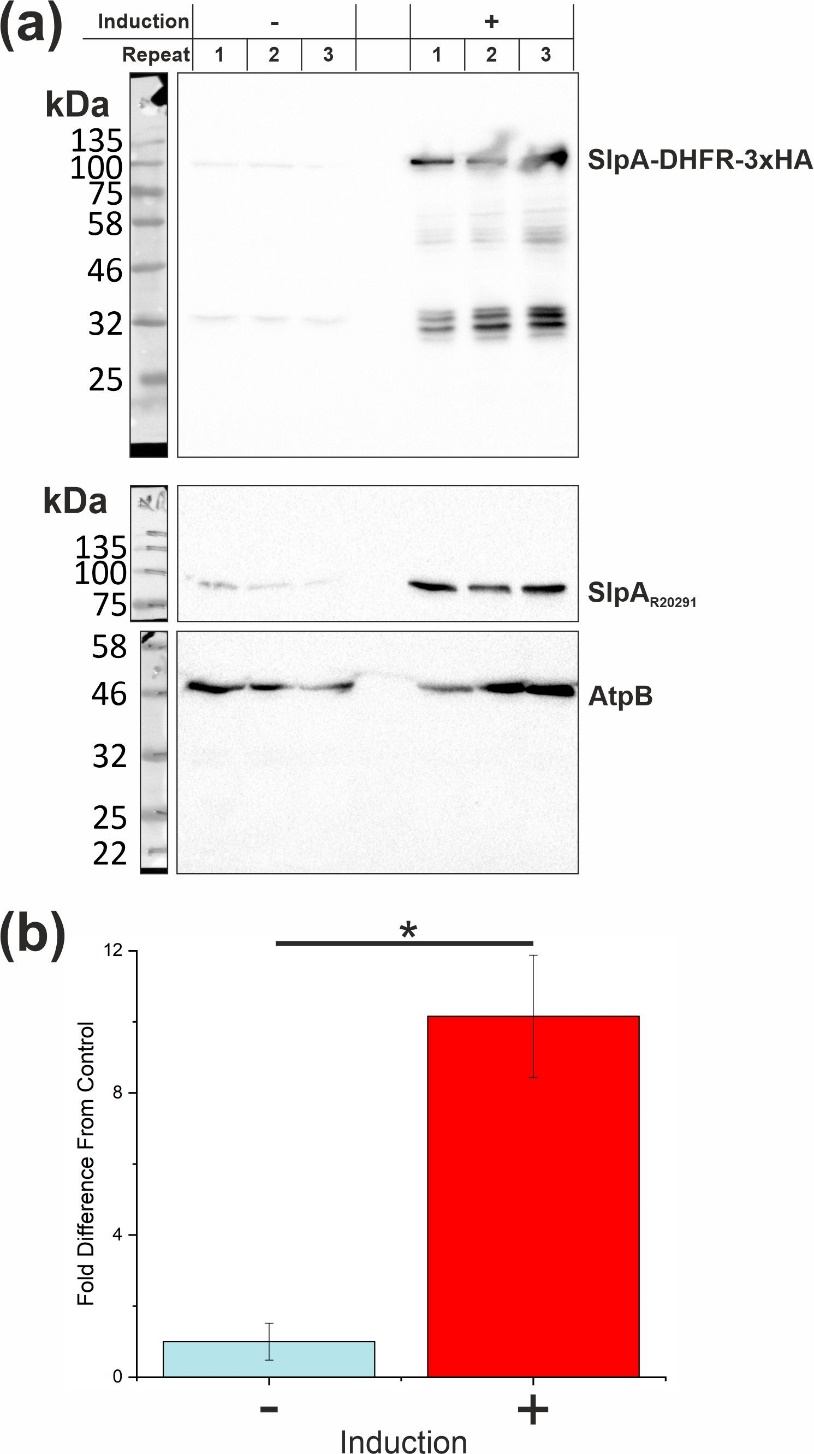


#### Supplementary Figure 6: Characterization of a SlpA-DHFR fusion protein

**(a)** Western immunoblot analysis of *C. difficile* R20291 expressing an SlpA_630_-DHFR-3xHA fusion. Protein expression was induced (+) with 20 ng/ml Atc for 1 hour and intracellular cell extracts (membrane and cytosol) were analyzed by SDS PAGE followed by Western immunoblot using an anti-HA antibody to show expression of SlpA_630_-DHFR-3xHA (top panel), anti-SlpA_R20291_ to visualize accumulation of native SlpA precursor in the cytosol (middle panel) and anti-AtpB as a membrane protein loading control for SlpA_R20291_ (bottom panel). The middle and bottom panels are from a single gel. Following transfer to nitrocellulose, the membrane was cut horizontally and probed with primary antibodies as indicated. Samples from triplicate cultures are shown.

**(b)** Quantification of average fold change of intracellular SlpA_R20291_ precursor band intensity from (a), with standard error bars. Native SlpA_R20291_ secretion is blocked by expression of SlpA_630_-DHFR-3xHA. One-way ANOVA analysis identified a significant difference in mean values *p <0.05).


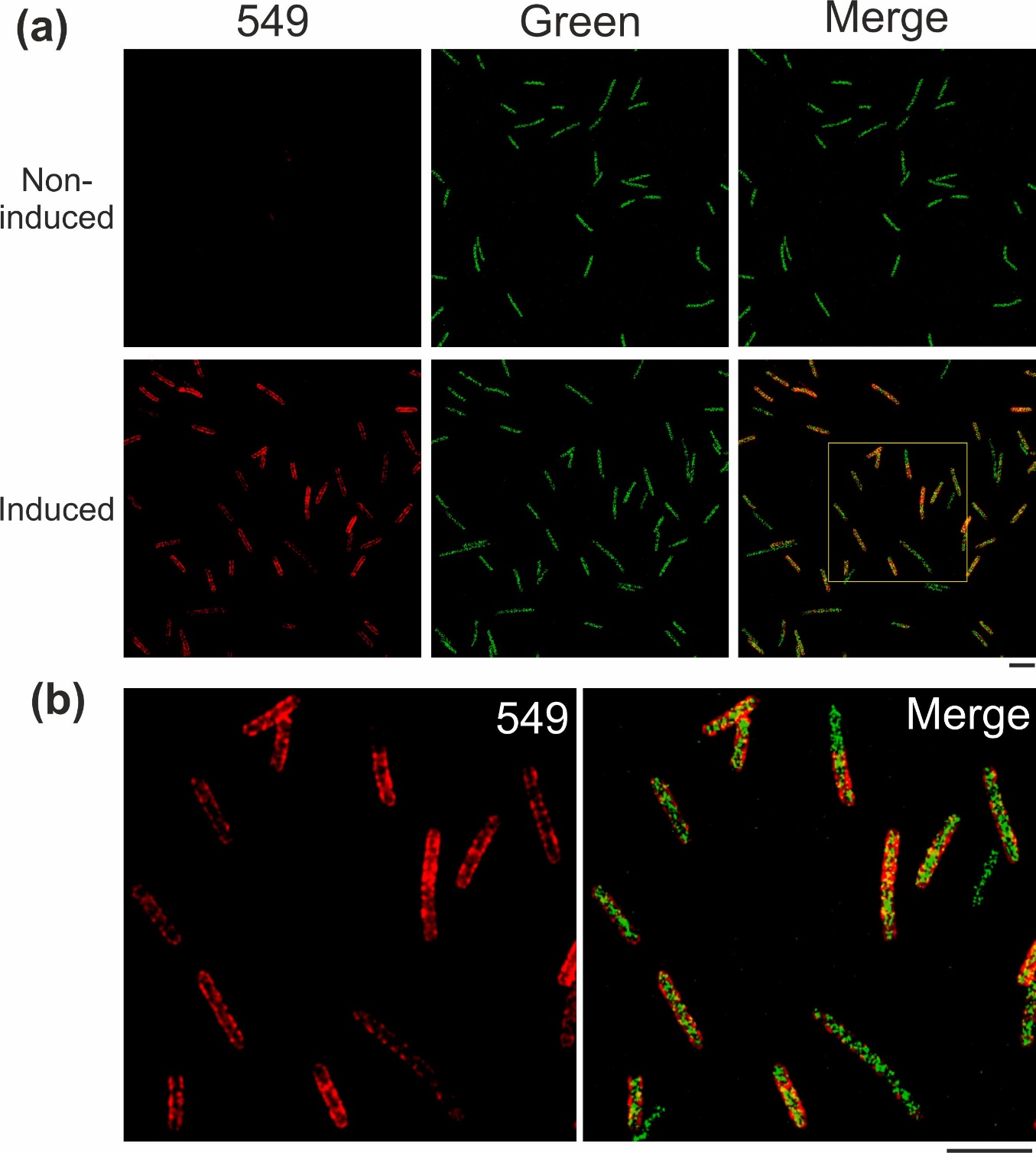


#### Supplementary Figure 7: SNAP-Surface 549 Stained HMW-SlpA-SNAP.

**(a)** Airyscan confocal images of *C. difficile* 630 cells stained with SNAP-Surface 549 and induced or not induced for SlpA_630_-SNAP expression. Surface 549 signal (left panels), green autofluorescence from *C. difficile* 630 cells (middle panels) and merged (right panels). Area taken for zoomed image depicted by a yellow square. Scale bar indicates 6 µm.

**(b)** Zoomed area (from (a)) of HMW-SLP-SNAP-Surface 549 signal (left panel) and autofluorescence merged image (right panel). Scale bar indicates 6 µm.


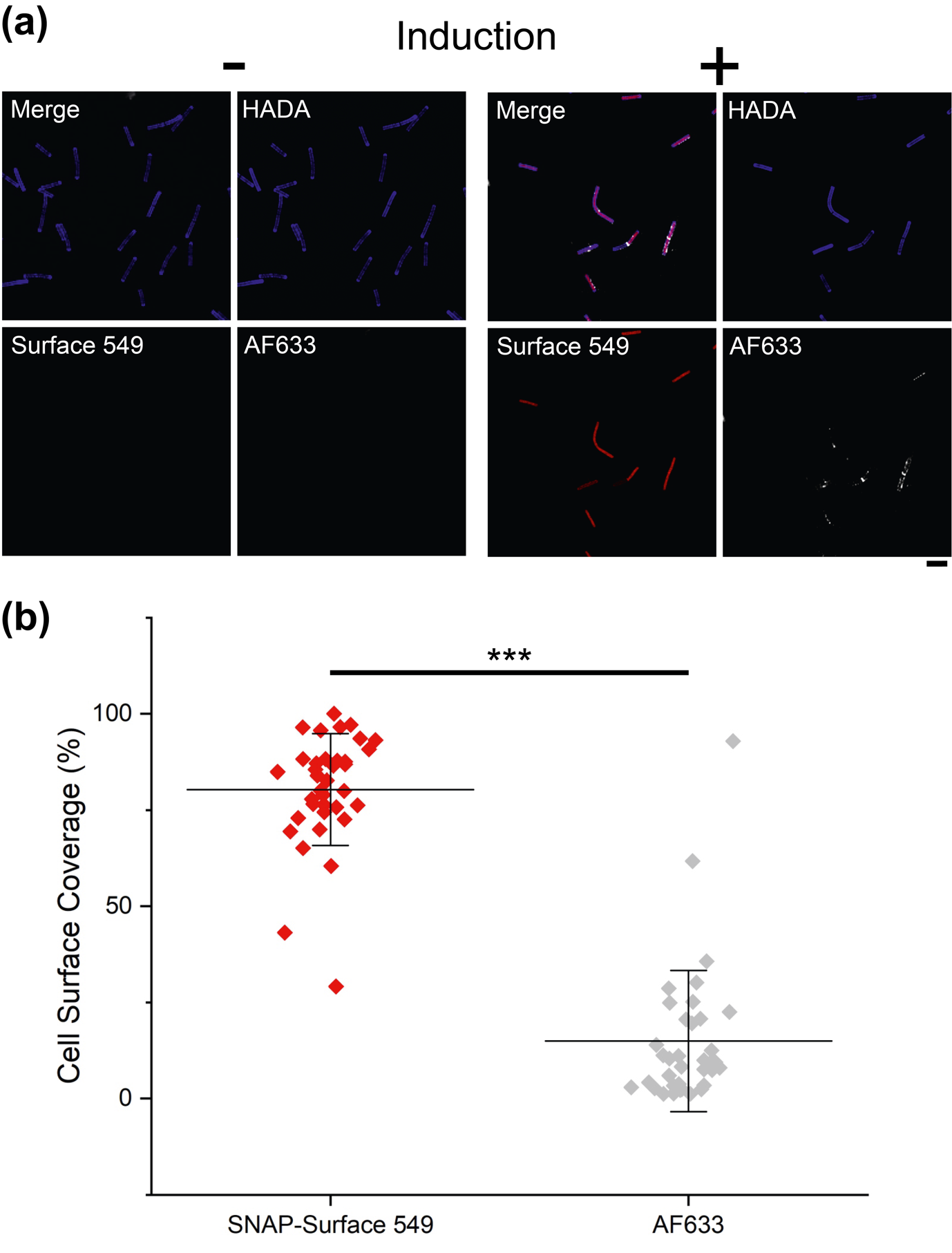
­

**Supplementary Figure 8: Surface localization of SlpA-SNAP.**

**(a)** Airyscan confocal imaging of *C. difficile* R20291 cells induced (+) or uninduced (-) for SlpA_630_-SNAP expression. Cells were simultaneously stained with SNAP-Surface-549 (red), to label all extracellular SlpA_630_, and AF633 (white), to label that portion accessible to antibody on the cell surface.

**(b)** Graph displaying the proportion of the cell surface displaying SNAP-Surface-549 signal (total extracellular SlpA_630_-SNAP) or AF633 signal (SlpA_630_-SNAP accessible to antibody on the cell surface). Shown is the mean and standard deviation of 36 cells. One-way ANOVA analysis identified a significant difference in mean values ***p < 0.01.

**
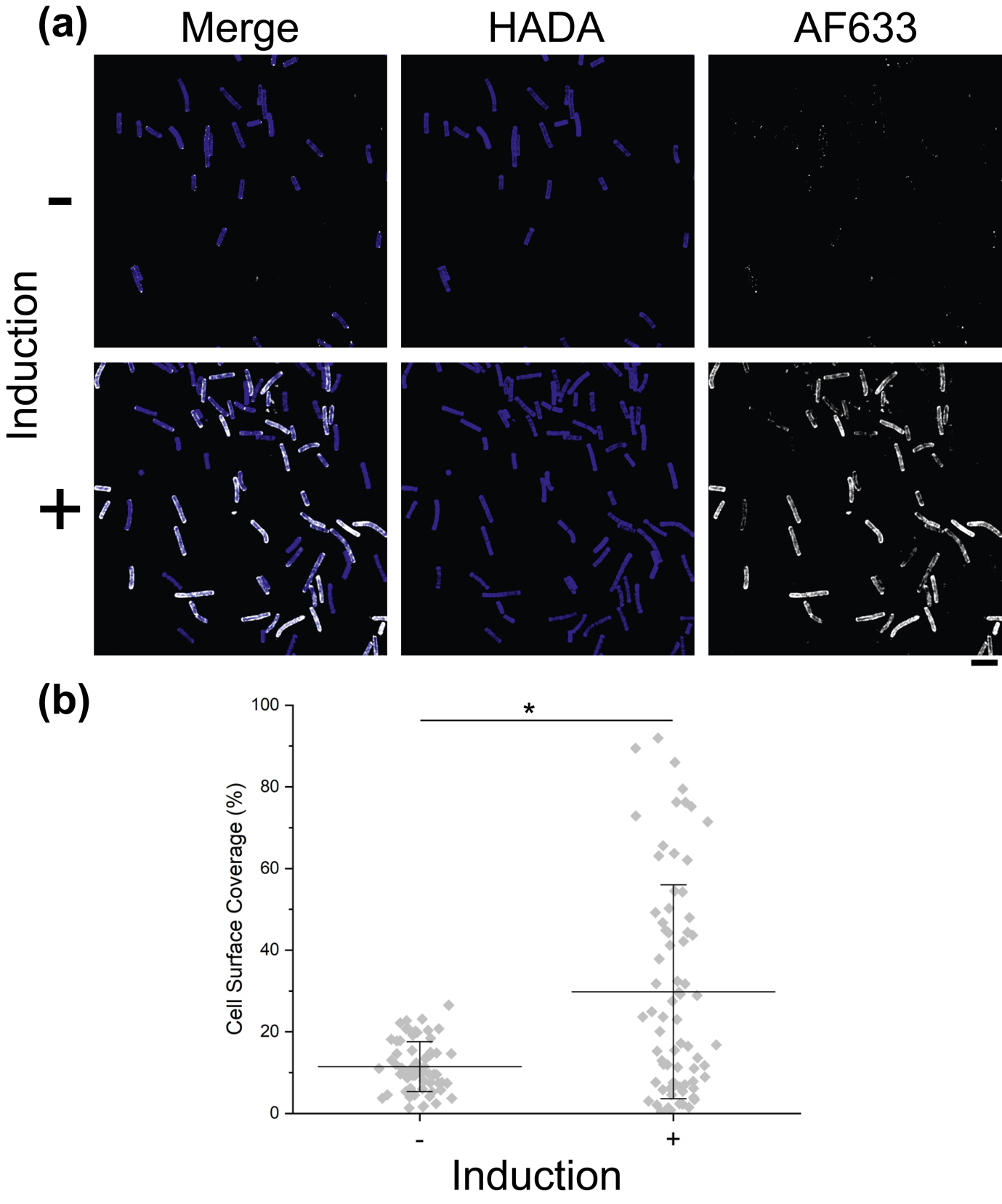
**

**Supplementary Figure 9: S-layer neogenesis in an S-layer deficient strain.**

**(a)** Airyscan confocal imaging of *C. difficile* FM2.5 cells induced (+) or uninduced (-) for SlpA_630_ expression with HADA signal (blue) and surface SlpA_630_ (LMW-SLP) labelled with AF633 (white).

**(b)** Surface coverage of FM2.5 cells with LMW_630_-SLP signal. The mean and stand deviation of measurements from 66 uninduced and 73 induced cells are shown. One-way ANOVA analysis identified a significant difference in mean values *p<0.05.

**Supplementary Table S1: Correlation of HADA and Cy5 signals for Figure 2.**

|  | Total | Significant | *p* = 0.01- 0.05 | *p* = 0.001 - 0.01 | *p* = 0.0001- 0.001 | *p* ≤ 0.0001 |
| --- | --- | --- | --- | --- | --- | --- |
| Negative correlation | 50 | 24 | 10 | 10 | 1 | 3 |
| Positive correlation | 26 | 6 | 1 | 3 | 0 | 2 |

Correlation analysis of HADA and Cy5 signal along the long edge of 76 cell sides as described in Figure 2 and Supplementary Figure 3. Cell long edges were classed as having a positive or negative HADA:Cy5 signal correlation and the significance levels of the significantly corelated signals are stated.

**Supplementary Table S2: Peak fitting analysis for Figure 5.**

|  | **Number of cells (%) with:** | | | |
| --- | --- | --- | --- | --- |
|  | **1 peak** | **2 peaks** | **More** | **Total** |
| **SlpA_630_-SNAP (TMR Star)** | 41 (59%) | 26 (38%) | 2 (3%) | 69 |
| **SlpA_630_-DHFR-SNAP (TMR Star)** | 41 (93%) | 3 (7%) | 0 (0%) | 44 |
| **SlpA_630_-SNAP (Surface)** | 2 (5%) | 33 (79%) | 7 (17%) | 42 |

Peak fitting analysis of cross-sectional SNAP-Cell TMR Star or SNAP-Surface 549 signal from *C. difficile* cells expressing the SNAP labelled forms of SlpA. The number of peaks was used to determine the localization of signal internally (1 peak) or at the cell periphery (2 peaks).

**Supplementary Table S3: Strains, plasmids and oligonucleotides used in this study**

| **Strain** | **Characteristics** | | **Source** |  |
| --- | --- | --- | --- | --- |
| R20291 | *C. difficile* ribotype 027 strain isolated during an outbreak at Stoke Mandeville hospital, UK in 2006 | | ^1^ |  |
| 630 | *C. difficile* ribotype 012 strain isolated during an outbreak in a hospital in Zurich, Switzerland in 1982 | | ^2^ |  |
| 630*secA2*-*snap* | *C. difficile* strain 630 with the sequence encoding SNAP added to the 3′ end of the *secA2* gene in the native locus | | This study |  |
| CA434 | *E. coli* strain CA434 (HB101 carrying R702) | | ^3^ |  |
| **Plasmid** | **Description** | | **Source** |  |
|  |  | |  | |
| pFT46 | P_tet_ *snap* | | ^4^ |  |
| pJAK014 | P_cwp2_ *secA2-snap* | | This study | |
| pJAK038 | P_tet_ *secA2-snap* | | This study |  |
| pJAK067 | pMTL-SC7315 modified to place *secA2*-*snap* on the *C. difficile* 630 chromosome | | This study |  |
| pJAK085 | P_tet_ *slpA_630_-hDHFR-myc-snap* | | This study |  |
| pMTL-SC7315 | Allele exchange vector | | ^5^ |  |
| pPOE002 | P_tet_ *slpA_630_-hDHFR-myc-strep tag II* | | This study |  |
| pPOE003 | P_tet_ *slpA_630_-hDHFR-myc-3xHA* | | This study |  |
| pPOE005 | P_tet_ *slpA_630-_strep tag II* | | This study |  |
| pPOE011 | P_tet_ *Δsignal sequence(ΔN2-A24)-slpA_630_-hDHFR-myc-strep tag II* | | This study |  |
| pPOE023 | P_tet_ *slpA_630_-snap* | | This study |  |
| pPOE032 | pMTL-SC7315 with *secA2-*(AEAAAKA) Linker*-snap* | | This study |  |
| pRPF173 | P_tet_ *slpA_630_*-*strep tag II* | | This Study |  |
| pRPF144 | P_cwp2_ *gusA* | | ^6^ |  |
| pRPF185 | P_tet_ *gusA* | | ^6^ |  |
| pRPF233 | P_tet_ *slpA_R20291_* | | ^7^ |  |
| pRPF238 | P_tet_ *slpA_R20291_*  *slpA_R20291_* modified such that the encoded LMW SLP contains a tetra cysteine motif (FLNCCPGCCMEP) in a predicted surface-exposed loop | | This study |  |
| **Oligo** | **Sequence** | **Use** | |  |
|  |  |  | |  |
| RF216* | GATCGAGCTCGGACAATAGAAAAGGAGGTACTTATATG | To amplify *secA2* with a 5' SacI site | |  |
| RF217* | GATCCTCGAGGTTAAATTTATATAAGTATTGCACTGTTGC | To amplify *secA2* with a 3' XhoI site | |  |
| RF218* | GATCCTCGAGGCAGCTGCTGATAAAGATTGTGAAATGAAGAGAACC | To amplify *snap* with a 5' XhoI site | |  |
| RF219* | GACTGGATCCAAGCTTTCCTTACCC | To amplify *snap* with a 3' BamHI site | |  |
| RF311 | TAGGGTAACAAAAAACACCG | Linearization of pMTL-SC7315 | |  |
| RF312 | CCTTTTTGATAATCTCATGACC | Linearization of pMTL-SC7315 | |  |
| RF411 | TTTTATTGCACTAGTTCCACCTG | Linearization of pRPF233 for insertion of the Tc encoding sequence | |  |
| RF412 | GATGTATTTGATACAGCTTTTACAG | Linearization of pRPF233 for insertion of the Tc encoding sequence | |  |
| RF635 | CGTAGAAATACGGTGTTTTTTGTTACCCTATCAATCTATAAATTAAATGTTGTCC | For Gibson assembly to place a *secA2*-*snap* homology cassette into pMTL-SC7315 | |  |
| RF636 | ATTACATGAACTTTTTTACCCAAGTCCTGGTTTC | For Gibson assembly to place a *secA2*-*snap* homology cassette into pMTL-SC7315 | |  |
| RF637 | CCAGGACTTGGGTAAAAAAGTTCATGTAATTTTTATTAAATG | For Gibson assembly to place a *secA2*-*snap* homology cassette into pMTL-SC7315 | |  |
| RF638 | GGGATTTTGGTCATGAGATTATCAAAAAGGCATATTACCTTTAACAGTTAATCTATATC | For Gibson assembly to place a *secA2*-*snap* homology cassette into pMTL-SC7315 | |  |
| RF721* | GTCACTCGAGGTTCGTCCGCTGAATTGTATTGTTGC | To amplify *DHFR* with a 5' XhoI site | |  |
| RF722* | GTCACTCGAGCAGATCTTCTTCGCTAATC | To amplify *DHFR* with a 3' XhoI site | |  |
| RF789 | GCAACTACTGGAACACAAG | To delete *slpA* signal peptide coding sequence | |  |
| RF790 | CATTTCTTAAATTCCTCCCAAC | To delete *slpA* signal peptide coding sequence | |  |
| RF811 | CCGGACTATGCAGGATCCTATCCATATGACGTTCCAGATTACGCTCCGTAAGGATCCTATAAGTTTTAATAAAAC | To add the triple HA tag coding sequence to *slpA-hDHFR* | |  |
| RF812 | GACGTCATAGGGATAGCCCGCATAGTCAGGAACATCGTATGGGTAAACCTCGAGCAGATCTTCTTC | To add the triple HA tag coding sequence to *slpA-hDHFR* | |  |
| RF866* | GATCGCGGCCGCCAGATCTTCTTCGCTAATCAGTTTC | To linearize pPOE002, adding a NotI site | |  |
| RF867 | CATCCACAATTTGAAAAATAAGGATCC | To linearize pPOE002, adding a NotI site | |  |
| RF868* | GATCGCGGCCGCTGATAAAGATTGTGAAATGAAGAGAACC | To amplify *snap* with a 5' NotI site | |  |
| RF869 | GTTACTAGTGGATCCAAGCTTTC | To amplify *snap* with a 5' NotI site | |  |
| RF1079 | TGCTAAGGCCGATAAAGATTGTGAAATGAAGAG | To change linker in pJAK067 (SecA2-SNAP in pMTL-SC7315) to AEAAAKA | |  |
| RF1080 | GCTGCCTCAGCGTTAAATTTATATAAGTATTGCACTG | To change linker in pJAK067 (SecA2-SNAP in pMTL-SC7315) to AEAAAKA | |  |

*Restriction endonuclease sites are underlined

#### Supplementary Movie 1: Live-cell imaging of *C. difficile* HADA chase.

Live-cell widefield microscopy of HADA fluorescent signal (top panel) and phase contrast (bottom panel) from *C. difficile* 630 cells chased for HADA staining, with a cell undergoing the final stages of cell division. 22 frames at ˜3 minutes/frame, scale bar indicates 3 µm.

**References**

1 Stabler, R. A. *et al.* Comparative genome and phenotypic analysis of *Clostridium difficile* 027 strains provides insight into the evolution of a hypervirulent bacterium. *Genome Biol* **10**, R102, doi:10.1186/gb-2009-10-9-r102 (2009).

2 Sebaihia, M. *et al.* The multidrug-resistant human pathogen *Clostridium difficile* has a highly mobile, mosaic genome. *Nat Genet* **38**, 779-786, doi:10.1038/ng1830 (2006).

3 Purdy, D. *et al.* Conjugative transfer of clostridial shuttle vectors from *Escherichia coli* to *Clostridium difficile* through circumvention of the restriction barrier. *Mol Microbiol* **46**, 439-452, doi:10.1046/j.1365-2958.2002.03134.x (2002).

4 Pereira, F. C. *et al.* The spore differentiation pathway in the enteric pathogen *Clostridium difficile*. *PLoS Genet* **9**, e1003782, doi:10.1371/journal.pgen.1003782 (2013).

5 Cartman, S. T., Kelly, M. L., Heeg, D., Heap, J. T. & Minton, N. P. Precise manipulation of the *Clostridium difficile* chromosome reveals a lack of association between the *tcdC* genotype and toxin production. *Appl Environ Microbiol* **78**, 4683-4690, doi:10.1128/AEM.00249-12 (2012).

6 Fagan, R. P. & Fairweather, N. F. *Clostridium difficile* has two parallel and essential Sec secretion systems. *J Biol Chem* **286**, 27483-27493, doi:10.1074/jbc.M111.263889 (2011).

7 Kirk, J. A. *et al.* New class of precision antimicrobials redefines role of C*lostridium difficile* S-layer in virulence and viability. *Sci Transl Med* **9**, doi:10.1126/scitranslmed.aah6813 (2017).
