# Supplementary figures and images for: Spatial organization of Clostridium difficile S-layer biogenesis
Source: Sci Rep. 2020 Aug 24;10:14089. doi: 10.1038/s41598-020-71059-x (PMC7445750; doi:10.1038/s41598-020-71059-x)

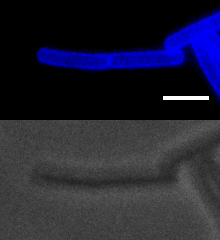

Supplement: Supplementary file 2 — Supplementary Movie. [file 41598_2020_71059_MOESM2_ESM.gif]
